# Photosynthesis is not the unique useful trait for discriminating salt tolerance capacity between sensitive and tolerant quinoa varieties

**DOI:** 10.1007/s00425-022-03928-w

**Published:** 2022-06-25

**Authors:** Aitor Agirresarobe, Jon Miranda-Apodaca, Iñaki Odriozola, Alberto Muñoz-Rueda, Usue Pérez-López

**Affiliations:** Departamento de Biología Vegetal y Ecología, Facultad de Ciencia y Tecnología, Universidad del País Vasco, UPV/EHU, Apdo. 644, 48080 Bilbao, Spain

**Keywords:** Phenotyping, Photosynthesis, Quinoa varieties, Salinity tolerance

## Abstract

**Main conclusion:**

**Growth was not strictly linked to photosynthesis performance under salinity conditions in quinoa. Other key traits, which were varieties-specific, rather than photosynthesis explained better growth performance**.

**Abstract:**

Phenotyping for salinity stress tolerance in quinoa is of great interest to select traits contributing to overall salinity tolerance and to understand the response mechanisms to salinity at a whole plant level. The objective of this work was to dissect the responses of specific traits and analyse relations between these traits to better understand growth response under salinity conditions in quinoa. Growth response to salinity was mostly related to differences in basal values of biomass, being reduced the most in plants with higher basal biomass. Regarding the relationship between growth and specific traits, in Puno variety, better photosynthetic performance was related to a better maintenance of growth. Nevertheless, in the rest of the varieties other traits rather than photosynthesis could better explain growth response. In this way, the development of succulence in F-16 and Collana varieties, also the osmotic adjustment but in smaller dimensions in Pasankalla, Marisma and S-15-15 helped to maintain better growth. Besides, smaller increases of Cl^−^ could have caused a limited nitrate uptake reducing more growth in Vikinga. Ascorbate was considered a key trait as a noticeable fall of it was also related to higher reductions in growth in Titicaca. These results suggest that, due to the genetic variability of quinoa and the complexity of salinity tolerance, no unique and specific traits should be taken into consideration when using phenotyping for analysing salinity tolerance in quinoa.

**Supplementary Information:**

The online version contains supplementary material available at 10.1007/s00425-022-03928-w.

## Introduction

Soil salinity is one of the most important environmental factors affecting yield (Abbas et al. 2021). Under soil salinity conditions, plants suffer from water deficit and ion toxicity. Due to the limitation of water availability and consequent water uptake, growth is affected by the reduction of CO_2_ availability resulting from stomatal closure and down regulation of photosynthetic metabolism (Jaramillo Roman et al. 2021). Under these conditions, plants may be also affected by the ionic component of salinity suffering ion toxicity (Munns and Tester 2008). At present, 20–50% of irrigated land is already salt affected (FAO 2021) due to natural conditions and inadequate agricultural management practices (Munns and Tester 2008). Besides, climate change can expand salt affected soils by increasing evapotranspiration and accumulating salts in the soils (Ullah et al. 2021). In consequence, suitable land for crop production is being reduced and thereby food production. Furthermore, human population is increasing exponentially so more food must be produced to satisfy the food demand (Panta et al. 2014).

Both factors, the yield loss and the increased food demand, apart from the fact that arable land is limited and is diminishing, make necessary to find new production strategies to guarantee crop production in salinity-affected areas. One of the approaches to face such situation is to increase crop diversification and introduce salt tolerant crops (Jacobsen 2003). Making traditional crops more tolerant to salinity may result difficult as the response to salinity includes several processes and mechanisms (Isayenkov 2012). Therefore, the use of naturally tolerant crops may result on a more cost-effective option (Zou et al. 2017).

Salinity tolerance comprises complex physiological responses to salt stress (Jaramillo Roman et al. 2021). In light of this, plant phenotyping helps on the dissection of such complex responses, analysing traits that contribute to overall salinity tolerance, which are more genetically tractable (Morton et al. 2019). Plant phenotyping is an important tool to address and understand plant environment interaction and its translation into application in crop management practices (Pieruschka and Schurr 2019). Although it has been considered that data sets of vegetative stage indoor experiments in grain crops have limited value for breeding, for prebreeding, the use of controlled-environment phenotyping is ideal to dissect physiological traits, analyse breeding progress, and generate genetic diversity with trait-based introgression (Watt et al. 2020). Phenotyping has been used under controlled environment conditions in some crops. Among those studies, water use strategies at early growth stages were studied in durum wheat (Nakhforoosh et al. 2016) and Avramova et al. (2016) performed a phenotyping for drought tolerance in maize. However, phenotyping was used for relatively few genotypes (Watt et al. 2020).

This approach may facilitate the search of suitable traits that explain the salinity tolerance of different varieties of the naturally tolerant quinoa. Quinoa is a facultative halophyte crop that originates from the Andean region in South America and it is an interesting alternative crop to be grown in salt affected areas, where the production of other traditional crops could be compromised (Hinojosa et al. 2018). Quinoa raised a big interest due to the high nutritional quality of its grain (Vilcacundo and Hernández-Ledesma 2017), but also because of its tolerance to some abiotic stresses, such as salinity (Bazile et al. 2016). Due to the great interest raised by this crop, new varieties adapted to different photoperiod and other environmental conditions have been developed to be cultivated in other parts of the world. It is the case of the salinity tolerant Danish bred cultivars Titicaca, Puno and Vikinga, or the recently selected varieties, such as Pin, F-16, S-15-15 and Marisma, in the south of Spain, where research and growth of quinoa is significantly increasing.

Although some salinity tolerance mechanisms have been described, quinoa shows a large genetic diversity that can provide different cultivars with a wide range of strategies to cope with salinity (Bonales-Alatorre et al. 2013; Bazile et al. 2016; Hinojosa et al. 2018). Nonetheless, few studies have investigated the response to salinity on a large scale of genotypes of quinoa (Gómez-Pando et al. 2010; Adolf et al. 2012; Shabala et al. 2013; Kiani-Pouya et al. 2019; Cai and Gao 2020). Along these studies, it has been concluded than Na^+^ loading capacity into the xylem, the stomatal density and the control of K^+^ homeostasis are important traits to explain salt tolerance, but also concluding that in some cultivars there is a trade-off between production and tolerance.

Few studies have analysed quinoa by a phenotyping approach although the potential of phenotyping to study salinity tolerance. For example, Gargiulo et al. (2019) evaluated different approaches to identify novel traits of quinoa grains useful for phenotyping and Jiang et al. (2022) evaluated the utility of unmanned aerial vehicles-based imagery to estimate leaf area index and chlorophyll content. Hinojosa et al. (2019) selected heat tolerant quinoa genotypes, among 112 accessions, for spectral reflectance indices, agronomic, and physiological trait evaluation under different irrigation regimes. However, still few studies analysed the salinity tolerance of this species by this approach. Besides, these studies analysed specific stages, such as the germination stage (Mizuno et al. 2020); specific measurements as yield components, ion concentrations and pigments (Rezzouk et al. 2020); or a few varieties (Jaramillo Roman et al. 2021). Considering this, phenotyping for salinity tolerance in quinoa taking into account different physiological processes and different varieties should be carried out.

Improving photosynthetic efficiency has recently emerged as a key strategy to increase yield potential of major crops (Zhu et al. 2020). Indeed, photosynthetic measurements have been described as a good phenotyping tool for breeding and for high precision crop management (Cruz and Avenson 2021). Even knowing the important role that photosynthetic rates play for biomass improvement (Becker et al. 2017) few studies have been done in quinoa correlating photosynthetic rates, biomass accumulation and salinity tolerance (González et al. 2011; Eisa et al. 2012; Hirich et al. 2014; Manaa et al. 2019). On the other hand, although in the aforementioned studies a positive correlation between photosynthetic rates and biomass accumulation was detected, the studies should analyse more cultivars, since those studies were done only in one or two cultivars in the same study. Besides, the assessment of photosynthesis and the relation with growth and other physiological processes such as ion homeostasis, water relations and antioxidant metabolism provides a mechanistic account of the salinity effects at a whole plant level (Jaramillo Roman et al. 2021).

So, knowing (1) the importance of the photosynthetic rates in explaining biomass accumulation, (2) that salinity tolerance is a multivariable component and (3) that genotype diversity helps to find a wide range of strategies to cope with salt stress, the aim of this paper is to study different physiological variables in several genotypes, trying to understand the relation of the photosynthetic performance with other salt tolerance variables for explaining biomass accumulation.

## Materials and methods

### Plant material and growing conditions

Nine quinoa (*Chenopodium quinoa* Willd.) varieties from different latitudes were used as plant material. The varieties Pasankalla and Collana originate from Puno region in Peru, the varieties Titicaca, Puno and Vikinga are Danish bred cultivars and the varieties Pin, F-16, S-15-15 and Marisma originate from Spain. Plant growth was carried out in an environmental controlled growth chamber under a daily regime of 14 h light—provided by warm-white (Philips TL5 HO 54 W/965) and cool-white (Osram HO 54 W/840) fluorescent lamps—and 10 h of darkness, with an average day/night temperature of 25/18 ºC and a relative humidity of 60/80%, respectively. Seeds were sown in a 3:1 mixture of perlite/vermiculite in 3L pots with 5 seeds per pot. Pots were thinned to one plant per pot 1 week after sowing. To minimize the effects of intrachamber environmental gradients, plants were repositioned randomly within the chamber each week.

Plants were watered with three times strengthen Hoagland´s solution one time per week and with distilled water two times per week until the beginning of the stress treatment, when the plants were 28 days. Stress treatment was imposed for 14 days, in which, plants were watered one time per week with 250 mL of three times strengthen Hoagland´s solution supplemented with a range of NaCl concentrations (0 mM = control, 100 mM, 200 mM, and 400 mM) and two times per week with distilled water supplemented with the same range of NaCl concentrations. Five biological replicates were used for each variety and treatment.

### Gas exchange parameters

Leaf gas exchange parameters were determined using a Li-6400 open gas exchange system (Li-Cor Inc., Lincoln, NE, USA). Measurements were done as Miranda-Apodaca et al. (2018). The photosynthetic photon flux density was 400 µmol m^−2^ s^−1^, provided by red/blue LED light source (model LI 6400-02B, Li-Cor Inc.). The CO_2_ concentration of the cuvette (*Ca*) was the same as in growth conditions (400 µmol mol^−1^ CO_2_). Li-Cor software was used to calculate stomatal conductance (*gs*), the intercellular CO_2_ concentration (*Ci*) and the net photosynthetic rate (*A*) according to the method of von Caemmerer and Farquhar (1981). The actual photochemical efficiency of photosystem II (*ɸPSII* = (*Fm*′–*F*s)/*Fm*′) was determined by measuring steady-state fluorescence (*Fs*) and maximum fluorescence during a light saturating pulse of 8000 µmol m^−2^ s^−1^ (*Fm*′) following the procedures of Genty et al. (1989).

### Growth parameters

At day 42, plants were separated into leaves, stems, and roots, and weighed to determine fresh weight (*FW*). The plant samples were dried at 80 ºC for 48 h and then the leaf, stem and root dry weights (*DW*), and total dry weight (*TDW*) were determined. Shoot to root *DW* ratio (*SRDW*) was expressed as the ratio between the sum of the aerial organs (leaf and stem) *DW* and the root *DW*. The leaf to stem *DW* ratio (*LSDW*) were determined as the ratio between the leaf and the stem *DW*.

### Plant water status parameters

The cumulative plant water transpiration (*Ctrans*) was calculated by gravimetric method. Each pot was weighed every 2 days at the same time, before and after watering (De Luis et al. 1999). The leaf osmotic potential (*OP*) was measured through the freezing point of the cellular sap by an osmometer (Osmomat 030, Gonotec, Germany) (Pérez-López et al. 2009a). The osmotic adjustment (*OA*) was calculated as Miranda-Apodaca et al. (2018). Whole plant water use efficiency (*WUE*) was calculated as the *TDW* divided by *Ctrans* (Pérez-López et al. 2014b). The relationship between leaf *FW* and *DW* (*FWDW*) was determined as the leaf *FW*/leaf *DW* ratio. The succulence of the leaves (*SUC*) was calculated as the *FW* of the leaves divided into the leaf area (*LA*), which was measured using an Epson expression 10000XL scanner.

### Mineral analysis

For mineral analysis finely grounded material samples from leaves, stems and roots was used. Cl^−^ concentration was determined in leaves, stems and roots by Qualitative Ionic Chromatography (Ionic Chromatograph Dionex ICS-5000+). For this, 0.1 g *DW* of grounded plant material was homogenised with 2 ml Mili-Q water. The homogenates were incubated at 90 ºC for 10 min and then centrifuged at 16,100 g for 5 min. The supernatant was filtered by a 0.2 μm 13 mm filter. For K^+^, Na^+^ and Ca^+2^ analysis, grounded plant material (0.5 g *DW*) was dissolved in 10 mL nitric acid (1%). The ions were determined with inductively coupled plasma atomic emission spectroscopy (ICP-AES, Horiba Jobin Yvon Activa). Cl^−^, Na^+^, K^+^ and Ca^+2^ uptake rates (*ClUR*, *NaUR*, *KUR* and *CaUR*) were measured as Pérez-López et al. (2014a). To indicate the potential of the translocation of ions from root to shoot, shoot to root ratios (*SRNa*, *SRCl*, *SRK*, *SRCa*) were measured as follows, where *M* was the mineral measured, leaf dry weight was indicated as *LDW*, stem dry weight as *SDW* and root dry weight as *RDW*:

$$\mathrm{SR}=\frac{\mathrm{LDW}\cdot \mathrm{leaf}\left[M\right]+\mathrm{SDW}\cdot \mathrm{stem}\left[M\right]}{\mathrm{RDW}\cdot \mathrm{root}\left[M\right]}.$$

To indicate the potential of the translocation of ions from stems to leaves, leaf to stem ratios (*LSNa*, *LSCl*, *LSK*, *LSCa*) were measured as follows, where *M* was the mineral measured:

$$\mathrm{LS}=\frac{\mathrm{LDW}\cdot \mathrm{leaf}\left[M\right]}{\mathrm{SDW}\cdot \mathrm{stem}\left[M\right]}.$$

### Antioxidant metabolism

Reduced ascorbate (*ASA*) and reduced glutathione (*GSH*) contents in leaves were determined according to Pérez-López et al. (2010). Catalase (*CAT*), superoxide dismutase (*SOD*), glutathione reductase (*GR*), monodehydroascorbate reductase (*MDHAR*) and dehydroascorbate reductase (*DHAR*) activities were basically measured according to Pérez-López et al. (2009b).

### Statistical analysis

To explore the nine quinoa varieties and their overall responses to the salinity gradient in terms of growth, water relations, photosynthesis, ion homeostasis and antioxidant capacity, we conducted an ordination of the samples through principal component analysis (PCA) on the correlation matrix of all measured physiological variables. These include *TDW, SRDW, LSDW, Ctrans, OP, OA, FWDW, SUC, WUE, A, gs, Ci, ɸPSII, NaUR, ClUR, KUR*, *CaUR, SRNa*, *SRCl, SRK, SRCa, LSNa, LSCl, LSK*, *LSCa, ASA, GSH, CAT, SOD, GR, DHAR* and *MDHAR*. We performed the PCA using the function rda() from the package vegan (Oksanen et al. 2020) in R (R Core Team 2021).

Then, we modelled the effect of salinity and variety on each physiological variables using linear models through the lm() function in R. Above physiological descriptors were used as response variables. Log transformations were applied using base e. As explanatory variables we included salinity treatment (fitted as a continuous variable), variety (with nine levels, S-15-15, Collana, F-16, Marisma, Pasankalla, Pin, Puno, Titicaca and Vikinga), and the interaction between salinity and variety. To allow nonlinear relationships with salinity, the quadratic term of salinity and the interaction with variety were also included in the models. To obtain parsimonious models, we dropped non-significant interaction terms by means of likelihood ratio tests.

## Results

### Principal component analysis

**Fig. 1:**
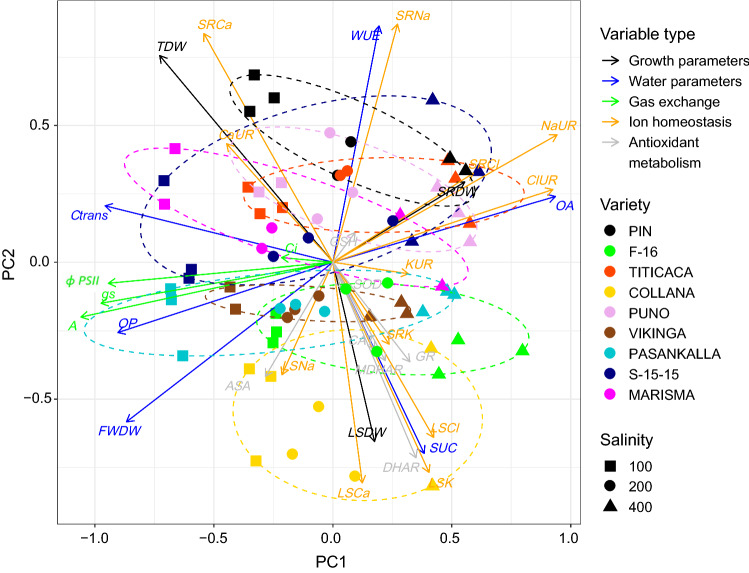
Principal component analysis (PCA) of the measured variables (grouped by physiological processes as: growth related parameters in black (*TDW, SRDW, LSDW*); plant water status parameters in blue (*Ctrans, OP, WUE, OA, FWDW, SUC*); gas exchange parameters in green (*A, gs, Ci, ɸPSII*); parameters related to ion homeostasis in orange (*NaUR, ClUR, KUR, CaUR, SRNa, LSNa, SRCl, LSCl, SRK, LSK, SRCa, LSCa*); antioxidant metabolism in grey (*ASA, GSH, CAT, GR, MDHAR, DHAR, SOD*) under different salinity treatments (as square for 100 mM NaCl, circle for 200 mM NaCl and triangle for 400 mM NaCl) in nine *Chenopodium quinoa* Willd. varieties (coloured as: dark blue: S-15-15; yellow: Collana; green: F-16; pink: Marisma; light blue: Pasankalla; black: Pin; light purple: Puno; red: Titicaca; brown: Vikinga). Projection length of the arrows indicate how much weight have each variable on the principal components. The angles between arrows indicate possible correlations between variables

Principal component analysis was performed to have an overall view of the obtained data and to analyse the relationships between variables (Fig. 1). PC1 divided salinity treatments by stress intensity. 100 mM NaCl treatment samples were located on the left side, 200 mM NaCl treatment samples in the centre and 400 mM NaCl treatment samples on the right side of the ordination. PC2 instead, separated varieties. PC1 represented a strong contrast between *OA*, *NaUR*, *ClUR*, *SRCl* and *SRDW*, all of them positively associated with salinity treatment, and, photosynthesis related parameters (*A*, *gs*, *Ci* and *ɸPSII*), *Ctrans* and *OP*, negatively associated with salinity. *WUE* and *SRNa* strongly and positively associated with PC2 and they were correlated with varieties such as Pin, S-15-15 and Puno. *LSCa*, *SUC*, *LSK*, *LSCl*, *LSDW* and *DHAR* associated negatively with PC2 and thus were correlated with the varieties placed in the bottom of the ordination, such as Collana.

### Gas exchange parameters

**Fig. 2:**
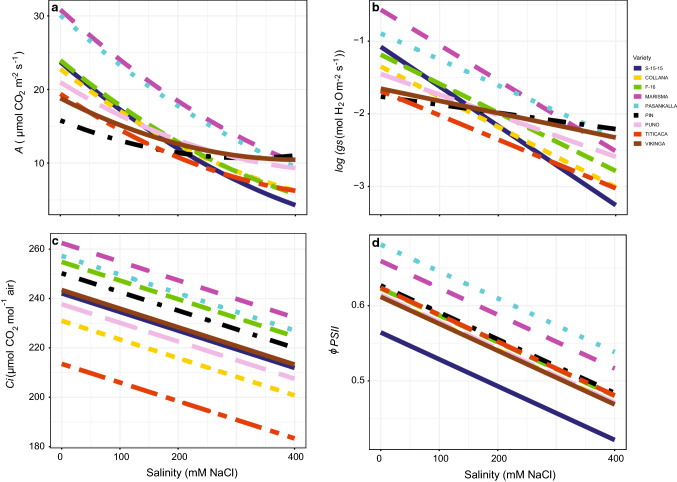
Photosynthetic rate (*A*, **a**), stomatal conductance (*gs*, **b**), intercellular CO_2_ concentration (*Ci*, **c**) and actual photochemical efficiency of photosystem II (*ɸPSII*, **d**) responses to different salinity concentrations (0, 100, 200, 400 mM NaCl) in the nine *Chenopodium quinoa* Willd. varieties indicated by different colour lines (continuous dark blue: S-15-15; discontinuous yellow: Collana; discontinuous green: F-16; discontinuous pink: Marisma; dotted light blue: Pasankalla; dotted and discontinuous black: Pin; discontinuous light purple: Puno; discontinuous red: Titicaca; continuous brown: Vikinga). Values represent mean values of 5 plants per treatment

Salinity and variety affected significantly (*P* < 0.05; Table S1) the measured photosynthesis related variables (*A*, *gs*, *Ci* and *ɸPSII*) (Fig. 2, Table S2), being all reduced by salt treatment. Besides, for *A* and *gs* a significant interaction between salinity and variety was observed (*P* < 0.05; Table S1). Marisma and Pasankalla showed the highest values of *A* (Fig. 2a) and *gs* (Fig. 2b) under non-saline conditions. In contrast, the lowest values were obtained in Pin, Vikinga and Titicaca. Precisely the biggest reductions of *A* and *gs* were obtained in Marisma, Pasankalla and S-15-15 while the lowest in Pin and Vikinga. Moreover, in the case of *A*, the response curves were stabilized between 200 and 400 mM NaCl conditions only in Pin and Vikinga. The responses to salinity did not vary among varieties for *Ci* (Fig. 2c) and *ɸPSII* (Fig. 2d).

### Growth parameters

**Fig. 3:**
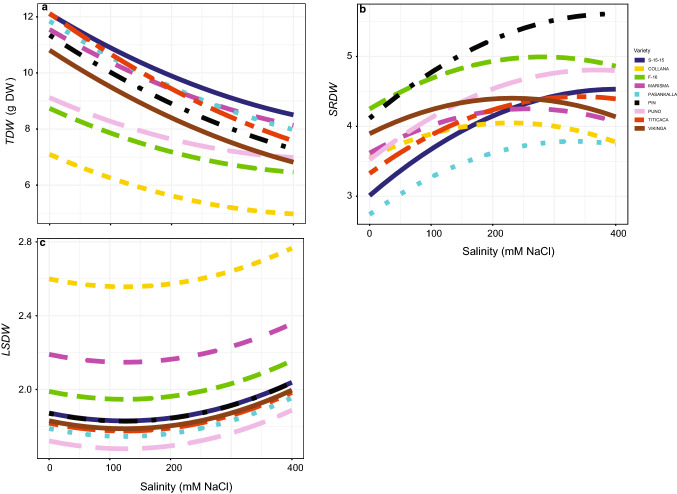
Total dry weight (*TDW*, **a**), shoot-to-root dry weight ratio (*SRDW*, **b**) and leaf-to-stem dry weight ratio (*LSDW*, **c**) responses to different salinity concentrations in the nine *Chenopodium quinoa* Willd. varieties (salt concentrations, line colouration for each variety and number of samples per treatment are as in Fig. 2)

Salinity decreased growth (Table S3) by reducing *TDW* (Fig. 3a). By contrast, the other two growth related variables analysed (*SRDW* and *LSDW*) increased with salinity (*P* < 0.05; Table S1) (Fig. 3b, c). Varieties could be divided into three groups by the values of *TDW* obtained under non-saline conditions. In this way, S-15-15, Titicaca, Pasankalla, Marisma, Pin and Vikinga were the ones showing the highest values, Puno and F-16 were in the middle and Collana was the variety showing the smallest *TDW*. Significantly, the biggest reductions due to salinity were obtained in the varieties showing higher *TDW* under non-saline conditions. Among these, the biggest reductions were observed in Titicaca, Pin and Vikinga. It was observed that Pasankalla and S-15-15 showed clearly lower values of *SRDW* under non-saline conditions. Besides, responses of *SRDW* to salinity varied among varieties (*P* < 0.05; Table S1). Regarding *LSDW*, no interaction between salinity and variety was observed but varieties did vary in their basal values (*P* < 0.05; Table S1).

### Plant water status parameters

**Fig. 4:**
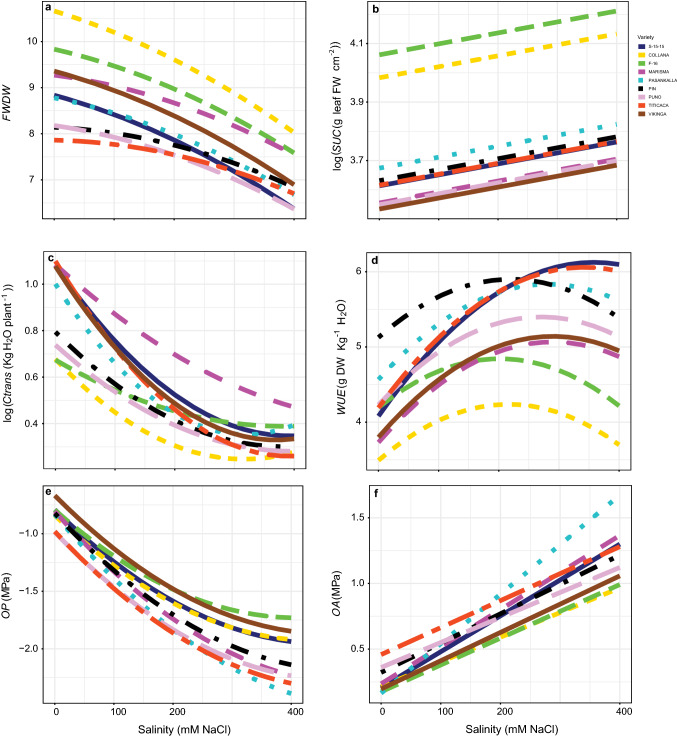
Fresh weight dry weight ratio (*FWDW*, **a**), succulence (*SUC*, **b**), cumulative transpiration (*Ctrans*, **c**), water use efficiency (*WUE*, **d**), osmotic potential (*OP*, **e**) and osmotic adjustment (*OA*, **f**) responses to different salinity concentrations in the nine *Chenopodium quinoa* Willd. varieties (salt concentrations, line colouration for each variety and number of samples per treatment are as in Fig. 2)

The interaction between salinity and variety was significant for almost all the variables related to the water relations (*P* < 0.05; Tables S1 and S4). The highest values of *FWDW* under non-saline conditions were obtained in Collana and F-16, and the lowest were obtained in Titicaca, Pin and Puno (Fig. 4a). *FWDW* correlated with *SUC* (data not shown); hence, the highest values of *SUC* were also obtained in F-16 and Collana (Fig. 4b). The reduction of *FWDW* by salinity depended on variety (*P* < 0.05). By contrast, *SUC* increased with salinity consistently between varieties.

Marisma, Titicaca, S-15-15, Vikinga and Pasankalla showed the highest values of *Ctrans* under non-saline conditions (Fig. 4c). *Ctrans* was reduced by salinity in all the varieties but bigger reductions were observed in the varieties starting at higher values. Besides, a linear reduction of *Ctrans* in Marisma was observed, while the response for the rest of the varieties was curvilinear. Those non-linear responses tended to stabilize above 200 mM NaCl concentration. Related to that, *WUE* increased exponentially with salinity in all the varieties but the highest increase was observed in S-15-15 and Titicaca (Fig. 4d). In contrast, the lowest values of *WUE* were obtained in F-16 and Collana. In addition, the response curves of *WUE* tended to stabilize in almost all the varieties.

Similar values of *OP* were observed for most of the varieties under non-saline conditions (Fig. 4e). However, their responses to salinity clustered in two groups: salinity decreased *OP* most in Pin, Puno, Marisma, Titicaca and Pasankalla. *OA* increased differentially with salinity in the varieties tested (*P* < 0.05) showing the highest increases in Pasankalla, Marisma and S-15-15 (Fig. 4f).

### Ion homeostasis

**Fig. 5:**
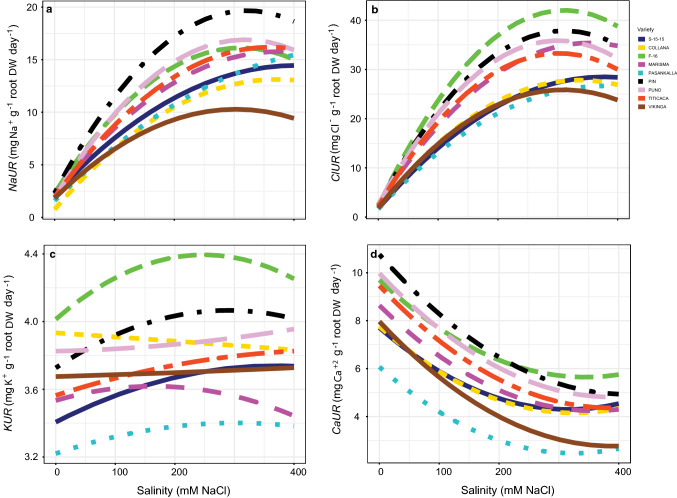
Uptake rates (*UR*) of the four nutrients analysed (*NaUR*, **a**; *ClUR*, **b**; *KUR*, **c**; *CaUR*, **d**) responses to different salinity concentrations in the nine *Chenopodium quinoa* Willd. varieties (salt concentrations, line colouration for each variety and number of samples per treatment are as in Fig. 2)

The interaction between salinity and variety was significant for the uptake rates (*UR*) of the four nutrients analysed (*P* < 0.05; Table S1) (Fig. 5, Table S5). Salinity increased *NaUR* in all the varieties (Fig. 5a). However, the response curves showed a tendency to stabilize in all the varieties. Pasankalla was the exception to this as *NaUR* increased linearly with salinity. The larger increase was obtained in Pin, whereas the lowest in Vikinga. In the same way, *ClUR* also increased with salinity (Fig. 5b). Stabilization tendencies were observed in most varieties but not in Marisma, Pasankalla and S-15-15. In contrast to Na^+^ and Cl^−^ uptake rates, *CaUR* decreased with salinity (Fig. 5d). The highest reductions of this variable were observed in Pin, Titicaca and Vikinga. In addition, *CaUR* tended to stabilize above 200 mM NaCl concentration. The highest values of *KUR* under non-saline conditions were obtained in F-16, Collana and Puno (Fig. 5c). *KUR* increased with salinity in the majority of the varieties, except in Collana and Marisma in which it was reduced, and it was maintained in Vikinga. Among the positive responses to salinity, the biggest increase was obtained in F-16. In comparison, the increases for *KUR* were much smaller than those observed for *NaUR* and *ClUR*.

**Fig. 6:**
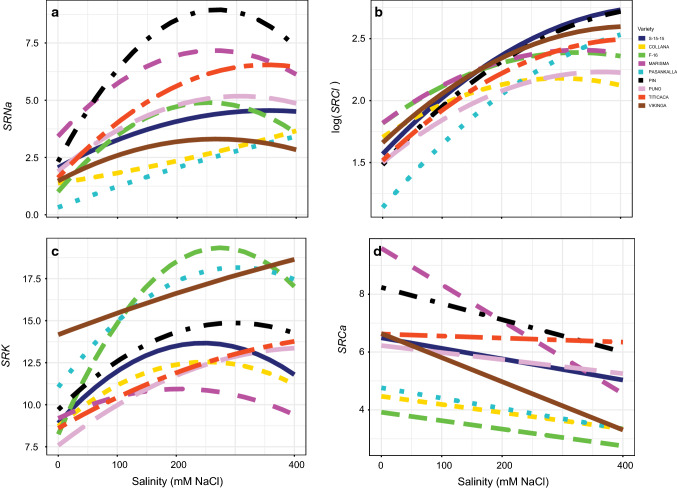
Shoot-to-root ratios (*SR*) of the four nutrients analysed (*SRNa*, **a**; *SRCl*, **b**; *SRK*, **c**; *SRCa*, **d**) responses to different salinity concentrations in the nine *Chenopodium quinoa* Willd. varieties (salt concentrations, line colouration for each variety and number of samples per treatment are as in Fig. 2)

There was a significant interaction between salinity and variety (*P* < 0.05; Table S1) in the shoot-to-root ratio (*SR*) of the nutrients analysed (Fig. 6, Table S6). The variables *SRNa*, *SRCl* and *SRK* increased with salinity. Regarding *SRNa*, the biggest increase was obtained in Pin (Fig. 6a). The smallest increases instead were obtained in F-16, Collana, Puno, S-15-15 and Vikinga. Besides, the response curves tended to stabilize above 200 mM NaCl concentration in some varieties. The biggest increases of *SRCl* were obtained in Pasankalla, Titicaca, Vikinga, Pin and S-15-15 (Fig. 6b). In respect of *SRK*, we observed that the response was linear in Vikinga but curve in the rest of the varieties (Fig. 6c). Among them, the biggest increases were obtained in F-16 and Pasankalla. In the majority of the varieties, the response curves of *SRK* tended to stabilize above 200 mM NaCl concentration. The response of *SRCa* was linear and declined with salinity in all the varieties (Fig. 6d).

**Fig. 7:**
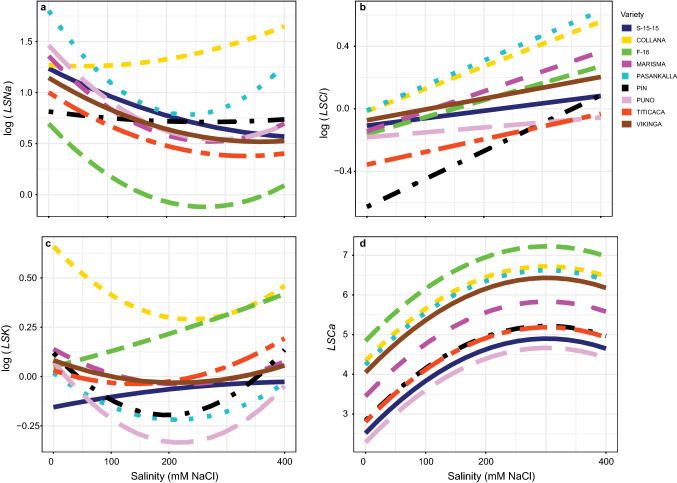
Leaf-to-stem ratios (*LS*) of the four nutrients analysed (*LSNa*, **a**; *LSCl*, **b**; *LSK*, **c**; *LSCa*, **d**) responses to different salinity concentrations in the nine *Chenopodium quinoa* Willd. varieties (salt concentrations, line colouration for each variety and number of samples per treatment are as in Fig. 2)

The salinity also affected significantly (*P* < 0.05; Table S1) the leaf-to-stem ratio (*LS*) in all the mineral elements studied (Fig. 7, Table S7), Na^+^, Cl^−^ and K^+^ showing significant interaction between salinity and variety (*P* < 0.05). *LSNa* response varied strongly among varieties (Fig. 7a). It was noticeable that responses of *LSNa* tended to stabilize above 200 mM NaCl in Pasankalla, F-16 and Puno. The response for K^+^ also varied strongly among varieties (Fig. 7c). *LSK* was increased in almost all the varieties except in Collana, Vikinga and Marisma. Regarding *LSCl*, it increased linearly with salinity but differently in all the varieties (Fig. 7b). No interaction between salinity and variety was detected for *LSCa* (Fig. 7d).

### Antioxidant metabolism

**Fig. 8:**
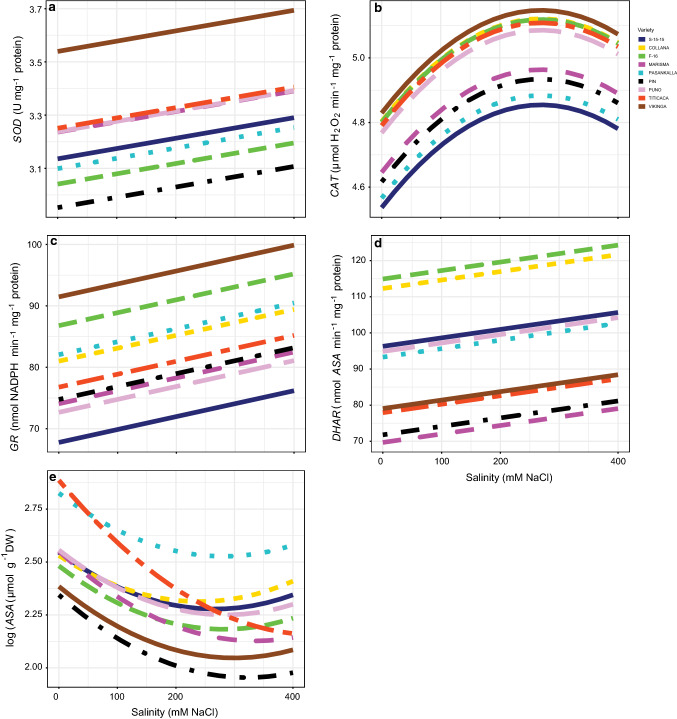
Superoxide dismutase (*SOD*, **a**), catalase (*CAT*, **b**), glutathione reductase (*GR*, **c**), dehydroascorbate reductase (*DHAR*, **d**) and reduced ascorbate (*ASA*, **e**) responses to different salinity concentrations in the nine *Chenopodium quinoa* Willd. varieties (salt concentrations, line colouration for each variety and number of samples per treatment are as in Fig. 2)

Salinity affected significantly (*P* < 0.05; Table S1) four (*CAT, SOD, DHAR* and *GR* activities) of the five antioxidant enzymes measured (Fig. 8, Table S8), as well as *ASA* (Fig. 8e). *SOD* and *CAT* increased with salinity but the response was linear for the first (Fig. 8a) while curvilinear for the second (Fig. 8b). The highest values of these two activities were obtained in Vikinga; very clearly in the case of *SOD*, and closely followed by F-16, Collana, Titicaca and Puno in the case of *CAT*. Furthermore, we observed that *CAT* increase was limited when 200 mM NaCl conditions were reached. Both *GR* and *DHAR* increased with salinity (Fig. 8c, d). As for *SOD* and *CAT*, the highest values of *GR* were obtained in Vikinga. For *DHAR*, the highest values were obtained in F-16 and Collana. Salinity had no effect neither on *MDHAR* activity, nor on *GSH* content (Table S8), showing maximum basal values in Puno variety. In contrast, *ASA* decreased with salinity differently in all the varieties, Titicaca showing the most marked reduction. Thus, *ASA* was the unique variable related to antioxidant metabolism showing a significant interaction between salinity and variety. It was also observed that the decrease in *ASA* with salinity tended to stabilize in almost all the varieties except in Titicaca.

## Discussion

Through this discussion, we will address the importance of photosynthetic rates in explaining biomass accumulation and we will also analyse other salt tolerance mechanisms, such as succulence, osmotic adjustment, ion homeostasis and antioxidant metabolism, using nine quinoa varieties from different origin. We will also try to elucidate if these physiological traits could be used for quinoa phenotyping.

Most of the varieties used in this study showed high photosynthesis rates under non-saline conditions. Adolf et al. (2013) suggested that higher values of photosynthesis rates and *gs* under non-saline conditions could be indicative of bigger productivity. Said this, although varietal differences were observed in the measurements of the variables related to photosynthesis, in principle, all of our varieties showed to be suitable candidates for a good production. The lowest value of *A* was observed in Pin, about 16 μmol CO_2_ m^−2^ s^−1^, which doubled the net photosynthetic rate obtained in Adolf et al. (2012) in Utusaya variety, considered to perform a trade-off strategy between productivity and tolerance.

Photosynthesis is among the most severely affected processes during salt stress exposure (Sudhir and Murthy 2004). Under salinity conditions, the osmotic component of the salt induces a stomatal closure, *Ci* declines at the same time that uptake and diffusion of CO_2_ to the RuBisCo carboxylation site is reduced and limits photosynthetic rates (Miranda-Apodaca et al. 2018). In fact, it is suggested that the osmotic component dominates plant response to salinity at the first days of salt imposition (Vita et al. 2021). Salt stress reduced *A* in our varieties. Similar values of assimilation rates of photosynthesis under salinity conditions to the ones obtained in this study were obtained by Becker et al. (2017). However, as we worked with different varieties, we observed significant differences in photosynthesis and stomatal conductance responses to salinity among them. It can be noticeable that the Danish bred cultivars (Vikinga, Puno and Titicaca) along with Pin were more capable of maintaining *A* rates under salinity conditions. Moreover, between 200 and 400 mM NaCl conditions the stabilization of the response curves observed in these varieties indicated that these varieties have mechanisms to keep photosynthetic machinery working under higher salinity conditions. In fact, those varieties showing the smallest reductions of *gs* (Vikinga, Pin and Puno) demonstrated to have a better capacity to maintain *A* under all salinity conditions indicating a smaller stomatal limitation. Waqas et al. (2019) also observed a correlation between *A* and *gs*.

Some authors have correlated photosynthetic rates, biomass accumulation and salinity tolerance (González et al. 2011; Eisa et al. 2012; Hirich et al. 2014; Manaa et al. 2019). In this study of nine varieties, this correlation was not detected for most of the varieties suggesting that vegetative growth in quinoa is not strictly linked to photosynthesis in most of the cases. On the contrary, those varieties showing a better maintenance of *A* and *gs* (Pin and Vikinga) showed bigger reductions in growth indicating that in these varieties other processes affected by salinity were also important for limitation of growth. Therefore, taking into account our results, the reduction of photosynthesis is not at least the mayor cause of growth reduction in the majority of the varieties. Exceptionally, in Puno, in concordance to the aforementioned studies, a better maintenance of *A*, along with a smaller reduction of *LA,* might be related to a better maintenance of growth under salinity.

The phenotyping approach permits to explore alternative individual traits as potential candidates for improving resistance to a given stress, but fast responses, such as stomatal conductance, might tend to overshadow slow but often more influential processes affecting growth (Passioura 2012). Considering this, other traits that take part in other physiological processes must be considered for their potential in phenotyping and to better explain quinoa growth response under salinity conditions at a whole plant level. Under salinity conditions, the mayor cause of biomass reduction could also be the loss of turgor (Munns and Tester 2008). Moreover, due to the complexity of salinity tolerance (Morton et al. 2019) and the ionic stress, other physiological processes such as ion homeostasis and antioxidant metabolism may also play a major role in salinity tolerance. This fact may affect the relationship between photosynthesis and growth.

Regarding water parameters, growth and productivity are often directly related to the amount of water transpired by the crop throughout its growth. Under salinity conditions, however, significant transpiration reductions occur in quinoa (Turcios et al. 2021). This strategy helps quinoa plants to reduce water loss (Razzaghi et al. 2011). The reduction of transpiration is related to both, reduction of *LA* and stomatal conductance. With respect to *LA* and consequently growth, our results showed that biomass production was strongly related to transpiration rate, also observed in González et al. (2011), and growth was also correlated positively with *LA* (data not shown). Higher basal values of Ctrans and bigger reductions of this variable were obtained in varieties showing larger biomass under non-saline conditions (Marisma, Vikinga, Titicaca, Pasankalla, S-15-15). In contrast, the smaller basal values of *Ctrans* and the smaller reductions of this variable were linked to varieties showing smaller biomass (Puno, F-16 and Collana). Therefore, having low biomass and *LA* could be an effective strategy to reduce water loss. However, Pin was clustered in the group of low reduction of *Ctrans* despite having high basal biomass and *LA*. That is, Pin could produce more biomass with less water as reflected by the observed high values of *WUE* under non-saline conditions. We think that the leaf in this variety may have a thicker boundary layer thus reducing the transfer of water vapour from the leaf to the environment. This may indicate that this variety had a bigger capacity to reduce water loss while maintaining stomata opened under salinity. However, in this case, it was not translated into smaller reductions in growth indicating that this plausible tolerance mechanism could be overshadowed by other mechanisms affecting growth.

Increasing succulence is another strategy to improve plant water status under saline conditions and it has been described in quinoa and other halophytes (Eisa et al. 2012; Razzaghi et al. 2015). However, varieties that were more succulent showed as big reductions of *A* as other varieties suggesting that the development of *SUC* could not be a key trait to keep photosynthesis and could be functioning with another objective. This might be in contrast with the idea that thicker leaves might increase chloroplast density thus maintaining *A* under salinity conditions (Munns and Tester 2008).

The development of succulence may also provide more space for an efficient sequestration of salinity related ions, such as Na^+^ (Shabala et al. 2013). In fact, under salinity conditions, quinoa decreases *OP* by accumulating inorganic ions, such as Na^+^, K^+^ and Cl^−^, while the metabolic cost required for the production of osmolytes is reduced (Ruiz et al. 2016a). In this way, quinoa plants rely on inorganic ions for osmotic adjustment to maintain turgor and water uptake from the salt affected soil (Razzaghi et al. 2015; Waqas et al. 2019). Pasankalla, Marisma and S-15-15 showed the most pronounced increases in inorganic ions with salinity. In the same time, they were among the varieties showing the most pronounced increases of *WUE*. This may suggest that the contribution of *OA* by means of accumulation of osmolytes could enhance water uptake and turgor maintenance, and thus support dry biomass production (Zhang et al. 2020) in Marisma, Pasankalla and S-15-15. The stronger increase of *OA* in these varieties could explain the advantage of them over the three varieties with the biggest reduction.

Moreover, the capacity of the most succulent varieties, F-16 and Collana, to maintain high amounts of *LSK* and higher basal values of *KUR* might indicate a relation between the development of *SUC* and K^+^ management to function in salinity tolerance. The efficient homeostatic mechanism present in quinoa includes a significant capacity to maintain high amounts of K^+^ that increase cytosolic osmolality (Adolf et al. 2013; Riccardi et al. 2014). Razzaghi et al. (2015) suggested that the capacity of increasing K^+^ under salinity was part of the extraordinary tolerance of quinoa to salinity. These two varieties showed also the lowest reductions in biomass. In light of this, we consider succulence performance as an important but energy costing mechanism that might be indicative of a trade-off between biomass accumulation and tolerance in F-16 and Collana.

Apart from the osmotic stress, plants grown under salinity conditions have to deal with ionic stress. In fact, under high salinity conditions Na^+^ and Cl^−^ can compete at root level with other nutrients, such as K^+^ and NO^−3^, respectively. In addition, salts may also accumulate in the apoplast and dehydrate the cell. Besides, if salt penetrates the cell, it can accumulate in the cytoplasm and cause the inhibition of the enzymes involved in carbohydrate metabolism (Munns and Tester 2008). In this sense, ion homeostasis plays a major role in the development, growth and tolerance of the plants under such stress conditions. In halophytes, salt tolerance is based on the inclusion and accumulation of salts in the aerial parts (Flowers and Colmer 2008). According to this, the salinity induced increase in the uptake rates and translocation to the shoot of Na^+^ and Cl^−^ observed in the varieties we tested suggested an inclusion strategy for these ions in quinoa species. This might affirm that most of the varieties we tested might rely on the use of these ions for osmotic adjustment. Although it is necessary to deal with excessive Na^+^ contents by limiting accumulation in active tissues as excessive Na^+^ contents are detrimental (Ruiz et al. 2016b), it seemed unlikely that Na^+^ builded-up and would have caused toxicity and non-stomatal limitations in our study as this occurs when tissue concentrations are above 250 mM (Munns et al. 2006). In light of this, Na^+^ concentrations in leaves were below the aforementioned concentration in all the varieties and treatments studied (data not shown) indicating that osmotic effects might have prevailed over ionic effects (Vita et al. 2021). For example, assimilation was reduced less in Pin despite showing the highest increase in both *NaUR* and *SRNa*, what probably increased in the same time the levels of Na^+^, indicating that photosynthesis was not affected by ion build-up, hence by toxicity (probably because the duration of salinity stress in our study was not long enough).

Chloride is an essential plant nutrient suggested to perform regulatory functions in photosynthesis, nutrition and growth (Li et al. 2017). Values of *ClUR* increased with salinity in all the varieties, being the increase of *ClUR* more prominent than that observed for *NaUR*. This difference could rely on the fact that chloride membrane transporters also transport nitrate (Böhm et al. 2018) and the latter must be uptaken continuously as it is essential for plant growth. In fact, in a previous paper, we hypothesized that quinoa regulates Cl^−^ uptake and indirectly NO_3_^−^ uptake (Miranda-Apodaca et al. 2020). This fact may reinforce the hypothesis that the lower values of *ClUR* obtained in Vikinga could imply lower nitrate uptake rates, explaining the higher growth reduction due to nitrogen limitation in this variety despite showing low reductions of *A*. On the other hand, Cl^−^ translocation to the leaves was enhanced with salinity indicating the need to accumulate Cl^−^ in the leaf vacuoles for osmotic adjustment purposes (Wu and Li 2019). Anyway, Cl^−^ has not been considered as important as Na^+^ in spite of being a major component of crop salt stress (Li et al. 2017). This underlines the necessity to deepen in research on chloride effects in plants and its importance in salinity tolerance.

High contents of Ca^+2^ in quinoa leaves contributing to osmotic adjustment have also been reported (Orsini et al. 2011). However, our results showed that *CaUR* rates decreased with salinity. This might be a clear sign that elevated salinity concentrations caused nutritional imbalance (Manishankar et al. 2018). We suggest that bigger reductions of *CaUR* could have affected the concentration of this ion in the apoplast and/or vacuoles and consequently, limit supply to the cytosol. In consequence, this might have limited stomatal closure, since it is known that the increase of calcium in the cytoplasm precedes stomatal closure (Ruiz et al. 1993) and might have contributed to an inadequate stomatal regulation in Pin, Vikinga, Titicaca and Puno. Considering this, however, we think that a major water loss, caused by a bigger reduction of *CaUR* linked to limited stomatal closure, might not be totally rejected in the aforementioned varieties. Evidently, more research must be done to study the effect of Ca^+2^ uptakes on stomatal conductance limitation under salinity conditions to check for this assumption. On the other hand, Ca^+2^ is also a signalling molecule. In that sense, the early cytoplasmic Ca^+2^ level oscillation mediated by the ROS-dependent Ca^+2^ channels, as well as the upregulation of the genes related to the abscisic acid (ABA) and ethylene response pathways, are significant traits to consider for the selection of salt-tolerant quinoa genotypes (Vita et al. 2021; Bazihizina et al. 2022).

The ABA produced in the roots is translocated to the leaves, where induces stomatal closure (Sun et al. 2014). This response induces reductions of *A* rates which may promote reactive oxygen species (ROS) production (Ruiz et al. 2016a). Halophytes, however, possess several efficient enzymatic and non-enzymatic antioxidant mechanisms to scavenge ROS and protect the cells from oxidative damage (Kumar et al. 2019). Due to the lack of consensus on the importance of the antioxidant metabolism in the salinity tolerance of quinoa, our results are of big interest. Among the studies that analysed antioxidant metabolism in quinoa under salinity conditions, Cai and Gao (2020) stated that *SOD*, *CAT* and *POD* were not important for salt tolerance, while Amjad et al. (2015) reported increased activities of such enzymes, along with ascorbate peroxidase (*APX*) activity, suggesting the antioxidant activity to be one of the factors responsible for quinoa salt tolerance. We did observe increases in four of the five enzymatic activities. However, the responses with respect to these activities were the same in all the varieties suggesting that antioxidant metabolism might not be a good indicator for discriminating salt tolerance capacity among varieties. Nevertheless, basal values varied strongly, what could explain differences in growth response. In this way, Puno showed clearly the highest values of *MDHAR* and *GSH,* which may be possibly linked to a better antioxidant activity and may support a better maintenance of growth under salinity. Regarding the non-enzymatic antioxidant mechanisms, *ASA* is an important ROS scavenger, being crucial for plant growth and development of abiotic stress tolerance (Xiao et al. 2021). Interestingly, it was the unique variable related to the antioxidant metabolism showing a significant interaction between salinity and variety. In this way, we observed a pronounced reduction of *ASA* in Titicaca. In this sense, we suggest that the decrease of *APX* activity in Titicaca could imply less H_2_O_2_ metabolized increasing damage and finally growth reduction.

## Conclusions

We conclude that the effect of salinity on vegetative growth was not strictly linked to photosynthesis performance under salinity conditions. Among the nine varieties tested, a better maintenance of growth could only be related to a better maintenance of *A* in Puno. In this way, it is necessary to take into account the contribution of other salt tolerance mechanisms to explain varietal different responses in final growth. Second, lower values of Cl^−^ were considered the most important variable affecting growth in Vikinga. Thirdly, stronger responses of OA allowed Marisma, Pasankalla and S-15–15 to maintain better turgor and obtain smaller reductions of growth. In addition, in Collana and F-16 more resources were invested for the performance of basal antioxidant components and for the development of larger succulence to favour tolerance at the expense of growth. Furthermore, higher basal values of antioxidants also favoured growth maintenance in Puno and the reduction of *ASA* was considered crucial for the reduction of growth in Titicaca. Finally, although different strategies contribute to the salinity tolerance, there are no key traits that could be used as unique criteria for the selection of quinoa varieties adapted to salt stress.

### *Author contribution statement*

AA, AM-R and UP-L conceived and designed research. AA, JM-A, AM-R and UP-L conducted the experiment and the analytical measurements. IO visualized data and conducted statistical analysis. AA, JM-A, AM-R and UP-L analysed data. AA wrote the first draft of the manuscript and all authors commented on previous versions. All authors read and approved the final manuscript.

## Supplementary Information

Below is the link to the electronic supplementary material.

Supplementary file1 (DOCX 23 KB)

Supplementary file2 (DOCX 73 KB)

## Data Availability

The authors declare that all the data supporting the findings of this study are available within the article [and its supplementary information files].
