# Supplementary material for: Photosynthesis is not the unique useful trait for discriminating salt tolerance capacity between sensitive and tolerant quinoa varieties
Source: Planta. 2022 Jun 25;256(2):20. doi: 10.1007/s00425-022-03928-w (PMC9233658; doi:10.1007/s00425-022-03928-w)
Supplement: Supplementary file 1 — Supplementary file1 (DOCX 23 KB) [file 425_2022_3928_MOESM1_ESM.docx]

| **Table 1** ANOVA tables of the models of the effect of variety (VAR), salinity (SALINITY) and their interaction (VAR:SALINITY) as well as the quadratic term of salinity and the interaction (Quadratic term) on each physiological variable grouped by physiological processes. Non-significant interaction terms were dropped by means of likelihood ratio tests and left blank spaces | **VAR** | | | **SALINITY** | | **SALINITY**  **(Quadratic term)** | | **VAR:SALINITY** | | **VAR:SALINITY (Quadratic term)** | |
| --- | --- | --- | --- | --- | --- | --- | --- | --- | --- | --- | --- |
|  | *F* | | *p* | *F* | *p* | *F* | *p* | *F* | *p* | *F* | *p* |
| **Gas exchange parameters** | |  |  |  |  |  |  |  |  |  |  |
| ***A*** | | 13.80 | <0.001 ^***^ | 458.82 | <0.001 ^***^ | 13.21 | <0.001 ^***^ | 8.04 | <0.001 ^***^ |  |  |
| ***gs*** | | 8.96 | <0.001 ^***^ | 325.42 | <0.001 ^***^ |  |  | 6.16 | <0.001 ^***^ |  |  |
| ***Ci*** | | 3.61 | < 0.001 ^***^ | 18.61 | < 0.001 ^***^ |  |  |  |  |  |  |
| ***ɸPSII*** | | 9.49 | < 0.001 ^***^ | 229.91 | < 0.001^***^ |  |  |  |  |  |  |
| **Growth parameters** | |  |  |  |  |  |  |  |  |  |  |
| ***TDW*** | | 36.86 | < 0.001^***^ | 229.05 | < 0.001 ^***^ | 5.30 | 0.02 ^*^ | 2.12 | 0.04 ^*^ |  |  |
| ***SRDW*** | | 21.22 | < 0.001 ^***^ | 78.48 | < 0.001 ^***^ | 22.58 | < 0.001 ^***^ | 3.25 | 0.002 ^**^ |  |  |
| ***LSDW*** | | 38.69 | < 0.001 ^***^ | 20.74 | < 0.001 ^***^ | 10.93 | 0.001 ^**^ |  |  |  |  |
| **Water status parameters** | |  |  |  |  |  |  |  |  |  |  |
| ***FWDW*** | | 58.18 | < 0.001 ^***^ | 542.32 | < 0.001 ^***^ | 12.97 | < 0.001 ^***^ | 4.12 | < 0.001 ^***^ |  |  |
| ***SUC*** | | 80.20 | < 0.001 ^***^ | 57.69 | < 0.001 ^***^ |  |  |  |  |  |  |
| ***Ctrans*** | | 46.03 | < 0.001 ^***^ | 1548.09 | < 0.001 ^***^ | 204.90 | < 0.001 ^***^ | 18.18 | < 0.001 ^***^ | 3.17 | 0.002 ^**^ |
| ***WUE*** | | 20.06 | < 0.001 ^***^ | 61.19 | < 0.001 ^***^ | 49.42 | < 0.001 ^***^ | 4.73 | < 0.001 ^***^ |  |  |
| ***OP*** | | 23.83 | < 0.001 ^***^ | 2080.14 | < 0.001 ^***^ | 101.26 | < 0.001 ^***^ | 5.46 | < 0.001 ^***^ |  |  |
| ***OA*** | | 15.52 | < 0.001 ^***^ | 673.97 | < 0.001 ^***^ |  |  | 5.10 | < 0.001 ^***^ |  |  |
| **Ion homeostasis** | |  |  |  |  |  |  |  |  |  |  |
| ***NAUR*** | | 14.30 | < 0.001 ^***^ | 950.81 | < 0.001 ^***^ | 169.04 | < 0.001 ^***^ | 3.60 | 0.001 ^**^ | 2.17 | 0.04 ^*^ |
| ***CLUR*** | | 12.78 | < 0.001 ^***^ | 903.29 | < 0.001 ^***^ | 237.65 | < 0.001 ^***^ | 2.46 | 0.02 ^*^ |  |  |
| ***KUR*** | | 121.05 | < 0.001 ^***^ | 34.64 | < 0.001 ^***^ | 20.91 | < 0.001 ^***^ | 5.25 | < 0.001 ^***^ | 5.11 | < 0.001 ^***^ |
| ***CAUR*** | | 36.74 | < 0.001 ^***^ | 577.38 | < 0.001 ^***^ |  |  | 3.35 | 0.002 ^**^ |  |  |
| ***SRNA*** | | 32.35 | < 0.001 ^***^ | 146.37 | < 0.001 ^***^ |  |  |  |  |  |  |
| ***SRCL*** | | 2.38 | 0.02 ^*^ | 141.90 | < 0.001 ^***^ | 12.88 | < 0.001 ^***^ | 2.53 | 0.02 ^*^ |  |  |
| ***SRK*** | | 15.77 | < 0.001 ^***^ | 67.26 | < 0.001 ^***^ | 29.00 | < 0.001 ^***^ | 2.39 | 0.02 ^*^ |  |  |
| ***SRCA*** | | 15.72 | < 0.001 ^***^ | 33.49 | < 0.001 ^***^ |  |  | 2.21 | 0.03 ^*^ |  |  |
| ***LSNA*** | | 19.71 | < 0.001 ^***^ | 38.08 | < 0.001 ^***^ | 30.81 | < 0.001 ^***^ | 3.12 | 0.004 ^**^ |  |  |
| ***LSCL*** | | 13.82 | < 0.001 ^***^ | 80.47 | < 0.001 ^***^ |  |  | 2.11 | 0.04 ^*^ |  |  |
| ***LSK*** | | 22.46 | < 0.001 ^***^ | 1.84 | 0.18 | 33.70 | < 0.001 ^***^ | 2.35 | 0.03 ^*^ | 2.19 | 0.04 ^*^ |
| ***LSCA*** | | 11.97 | < 0.001 ^***^ | 68.97 | < 0.001 ^***^ | 27.56 | < 0.001 ^***^ |  |  |  |  |
| **Antioxidant metabolism** | |  |  |  |  |  |  |  |  |  |  |
| ***CAT*** | | 5.25 | < 0.001 ^***^ | 17.96 | < 0.001 ^***^ | 18.08 | < 0.001 ^***^ |  |  |  |  |
| ***SOD*** | | 4.72 | < 0.001 ^***^ | 4.86 | 0.03 ^*^ |  |  |  |  |  |  |
| ***DHAR*** | | 18.87 | < 0.001 ^***^ | 7.53 | 0.007 ^**^ |  |  |  |  |  |  |
| ***GR*** | | 12.32 | < 0.001 ^***^ | 19.23 | < 0.001 ^***^ |  |  |  |  |  |  |
| ***MDHAR*** | | 8.82 | < 0.001 ^***^ |  |  |  |  |  |  |  |  |
| ***ASA*** | | 17.20 | < 0.001 ^***^ | 73.43 | < 0.001 ^***^ | 27.18 | < 0.001 ^***^ | 2.80 | 0.006 ^**^ |  |  |
| ***GSH*** | | 4.27 | < 0.001 ^***^ |  |  |  |  |  |  |  |  |
