# Supplementary material for: Photosynthesis is not the unique useful trait for discriminating salt tolerance capacity between sensitive and tolerant quinoa varieties
Source: Planta. 2022 Jun 25;256(2):20. doi: 10.1007/s00425-022-03928-w (PMC9233658; doi:10.1007/s00425-022-03928-w)
Supplement: Supplementary file 2 — Supplementary file2 (DOCX 73 KB) [file 425_2022_3928_MOESM2_ESM.docx]

**Table S2** Effects of salinity and variety on net photosynthetic rate (A; μmol CO_2_ m^-2^ s^-1^), stomatal conductance (gs; mol H_2_O m^-2^ s^-1^), intercellular CO_2_ concentration (Ci; μmol CO_2_ mol^-1^ air), and actual photochemical efficiency of photosystem II (ɸPSII). Var denotes variety; Salt, salinity treatment (0, 100, 200, 400 mM NaCl); Pin, Pin; F16, F-16; Tit, Titicaca; Col, Collana; Pun, Puno; Vik, Vikinga; Pas, Pasankalla; 15, S-15-15; Mar, Marisma. Mean values of five replicates ± standard error

| Var | Salt | *A* | | | *gs* | | | *Ci* | | | *ɸPSII* | | |
| --- | --- | --- | --- | --- | --- | --- | --- | --- | --- | --- | --- | --- | --- |
| Pin | 0 | 15.64 | ± | 1.37 | 0.21 | ± | 0.04 | 248.87 | ± | 15.42 | 0.60 | ± | 0.02 |
|  | 100 | 13.15 | ± | 1.22 | 0.15 | ± | 0.01 | 236.47 | ± | 4.22 | 0.58 | ± | 0.01 |
|  | 200 | 11.62 | ± | 1.04 | 0.12 | ± | 0.01 | 226.86 | ± | 6.53 | 0.54 | ± | 0.01 |
|  | 400 | 10.90 | ± | 0.42 | 0.12 | ± | 0.01 | 235.77 | ± | 15.77 | 0.54 | ± | 0.01 |
| F16 | 0 | 23.31 | ± | 0.87 | 0.27 | ± | 0.02 | 235.60 | ± | 8.53 | 0.64 | ± | 0.01 |
|  | 100 | 17.81 | ± | 1.47 | 0.25 | ± | 0.06 | 250.85 | ± | 15.65 | 0.59 | ± | 0.02 |
|  | 200 | 14.29 | ± | 1.65 | 0.16 | ± | 0.02 | 233.86 | ± | 4.53 | 0.57 | ± | 0.02 |
|  | 400 | 5.11 | ± | 0.78 | 0.06 | ± | 0.01 | 246.14 | ± | 21.89 | 0.45 | ± | 0.03 |
| Tit | 0 | 21.63 | ± | 1.17 | 0.26 | ± | 0.04 | 234.79 | ± | 17.88 | 0.65 | ± | 0.01 |
|  | 100 | 12.27 | ± | 1.78 | 0.11 | ± | 0.02 | 193.38 | ± | 11.45 | 0.56 | ± | 0.02 |
|  | 200 | 9.82 | ± | 1.16 | 0.08 | ± | 0.01 | 188.71 | ± | 5.05 | 0.53 | ± | 0.01 |
|  | 400 | 7.31 | ± | 0.60 | 0.06 | ± | 0.00 | 184.40 | ± | 5.09 | 0.51 | ± | 0.01 |
| Col | 0 | 22.86 | ± | 0.49 | 0.34 | ± | 0.10 | 236.52 | ± | 26.19 | 0.65 | ± | 0.02 |
|  | 100 | 17.35 | ± | 1.29 | 0.16 | ± | 0.02 | 202.95 | ± | 7.93 | 0.58 | ± | 0.02 |
|  | 200 | 12.00 | ± | 2.81 | 0.11 | ± | 0.02 | 212.96 | ± | 16.31 | 0.53 | ± | 0.03 |
|  | 400 | 6.46 | ± | 1.65 | 0.06 | ± | 0.01 | 218.60 | ± | 16.41 | 0.44 | ± | 0.04 |
| Pun | 0 | 20.65 | ± | 2.37 | 0.26 | ± | 0.06 | 237.25 | ± | 17.51 | 0.60 | ± | 0.02 |
|  | 100 | 15.56 | ± | 1.63 | 0.16 | ± | 0.02 | 226.37 | ± | 14.79 | 0.58 | ± | 0.01 |
|  | 200 | 15.22 | ± | 2.08 | 0.17 | ± | 0.03 | 232.14 | ± | 14.14 | 0.56 | ± | 0.02 |
|  | 400 | 8.52 | ± | 1.32 | 0.07 | ± | 0.01 | 202.18 | ± | 11.24 | 0.47 | ± | 0.03 |
| Vik | 0 | 18.94 | ± | 0.83 | 0.21 | ± | 0.01 | 238.31 | ± | 8.72 | 0.59 | ± | 0.01 |
|  | 100 | 14.87 | ± | 2.42 | 0.18 | ± | 0.03 | 241.76 | ± | 12.81 | 0.55 | ± | 0.03 |
|  | 200 | 12.89 | ± | 1.35 | 0.12 | ± | 0.01 | 213.32 | ± | 6.30 | 0.55 | ± | 0.01 |
|  | 400 | 10.41 | ± | 0.40 | 0.11 | ± | 0.01 | 227.51 | ± | 13.42 | 0.50 | ± | 0.01 |
| Pas | 0 | 29.17 | ± | 0.51 | 0.41 | ± | 0.03 | 262.23 | ± | 6.42 | 0.67 | ± | 0.00 |
|  | 100 | 26.26 | ± | 0.58 | 0.35 | ± | 0.05 | 251.94 | ± | 12.87 | 0.67 | ± | 0.00 |
|  | 200 | 15.33 | ± | 0.88 | 0.16 | ± | 0.02 | 223.96 | ± | 14.25 | 0.59 | ± | 0.01 |
|  | 400 | 9.98 | ± | 0.99 | 0.11 | ± | 0.01 | 237.99 | ± | 14.24 | 0.54 | ± | 0.02 |
| 15 | 0 | 23.52 | ± | 2.11 | 0.34 | ± | 0.05 | 264.08 | ± | 7.95 | 0.59 | ± | 0.03 |
|  | 100 | 19.02 | ± | 1.98 | 0.22 | ± | 0.04 | 228.51 | ± | 19.87 | 0.56 | ± | 0.02 |
|  | 200 | 9.92 | ± | 1.66 | 0.15 | ± | 0.06 | 240.48 | ± | 34.00 | 0.46 | ± | 0.03 |
|  | 400 | 4.91 | ± | 0.89 | 0.04 | ± | 0.01 | 182.49 | ± | 30.91 | 0.39 | ± | 0.02 |
| Mar | 0 | 29.21 | ± | 0.65 | 0.54 | ± | 0.04 | 288.14 | ± | 6.18 | 0.64 | ± | 0.00 |
|  | 100 | 25.82 | ± | 0.55 | 0.38 | ± | 0.05 | 267.99 | ± | 10.75 | 0.64 | ± | 0.00 |
|  | 200 | 19.14 | ± | 1.14 | 0.21 | ± | 0.01 | 239.20 | ± | 5.75 | 0.62 | ± | 0.01 |
|  | 400 | 9.30 | ± | 0.77 | 0.08 | ± | 0.01 | 201.75 | ± | 5.10 | 0.49 | ± | 0.01 |

**Table S3** Effects of salinity and variety on total dry weight (TDW; g DW), shoot to root dry weight ratio (SRDW), and leaf to stem dry weight ratio (LSDW). Var denotes variety; Salt, salinity treatment (0, 100, 200, 400 mM NaCl); Pin, Pin; F16, F-16; Tit, Titicaca; Col, Collana; Pun, Puno; Vik, Vikinga; Pas, Pasankalla; 15, S-15-15; Mar, Marisma. Mean values of five replicates ± standard error

| Var | Salt | *TDW* | | | *SRDW* | | | *LSDW* | | |
| --- | --- | --- | --- | --- | --- | --- | --- | --- | --- | --- |
| Pin | 0 | 11.18 | ± | 0.07 | 4.04 | ± | 0.24 | 1.93 | ± | 0.03 |
|  | 100 | 10.40 | ± | 0.44 | 4.85 | ± | 0.40 | 1.79 | ± | 0.05 |
|  | 200 | 8.70 | ± | 0.18 | 5.27 | ± | 0.18 | 1.89 | ± | 0.03 |
|  | 400 | 7.30 | ± | 0.52 | 5.58 | ± | 0.28 | 1.97 | ± | 0.05 |
| F16 | 0 | 8.92 | ± | 0.37 | 4.15 | ± | 0.24 | 2.02 | ± | 0.09 |
|  | 100 | 7.50 | ± | 0.78 | 4.59 | ± | 0.22 | 2.01 | ± | 0.08 |
|  | 200 | 7.36 | ± | 0.29 | 5.28 | ± | 0.42 | 1.92 | ± | 0.06 |
|  | 400 | 6.47 | ± | 0.49 | 4.71 | ± | 0.14 | 2.11 | ± | 0.12 |
| Tit | 0 | 12.36 | ± | 0.33 | 3.42 | ± | 0.11 | 1.72 | ± | 0.05 |
|  | 100 | 10.62 | ± | 0.12 | 3.77 | ± | 0.10 | 1.76 | ± | 0.06 |
|  | 200 | 9.03 | ± | 0.30 | 4.21 | ± | 0.22 | 1.84 | ± | 0.05 |
|  | 400 | 7.79 | ± | 0.18 | 4.43 | ± | 0.15 | 2.05 | ± | 0.06 |
| Col | 0 | 6.95 | ± | 1.01 | 3.67 | ± | 0.22 | 2.64 | ± | 0.16 |
|  | 100 | 6.38 | ± | 0.44 | 3.88 | ± | 0.08 | 2.52 | ± | 0.11 |
|  | 200 | 5.73 | ± | 0.42 | 3.82 | ± | 0.13 | 2.51 | ± | 0.16 |
|  | 400 | 4.89 | ± | 0.28 | 3.89 | ± | 0.11 | 2.82 | ± | 0.07 |
| Pun | 0 | 8.36 | ± | 0.88 | 3.65 | ± | 0.22 | 1.78 | ± | 0.20 |
|  | 100 | 8.94 | ± | 0.49 | 4.01 | ± | 0.07 | 1.62 | ± | 0.06 |
|  | 200 | 8.17 | ± | 0.72 | 4.44 | ± | 0.21 | 1.70 | ± | 0.09 |
|  | 400 | 6.55 | ± | 0.54 | 4.87 | ± | 0.21 | 1.88 | ± | 0.11 |
| Vik | 0 | 10.67 | ± | 0.21 | 4.06 | ± | 0.11 | 1.73 | ± | 0.06 |
|  | 100 | 9.69 | ± | 0.39 | 4.20 | ± | 0.21 | 1.86 | ± | 0.04 |
|  | 200 | 8.40 | ± | 0.28 | 4.12 | ± | 0.17 | 1.85 | ± | 0.04 |
|  | 400 | 6.77 | ± | 0.24 | 4.28 | ± | 0.22 | 1.98 | ± | 0.06 |
| Pas | 0 | 12.31 | ± | 0.52 | 2.57 | ± | 0.14 | 1.78 | ± | 0.07 |
|  | 100 | 10.12 | ± | 0.30 | 3.60 | ± | 0.19 | 1.76 | ± | 0.06 |
|  | 200 | 9.25 | ± | 0.41 | 3.46 | ± | 0.22 | 1.76 | ± | 0.07 |
|  | 400 | 8.21 | ± | 0.33 | 3.74 | ± | 0.16 | 1.95 | ± | 0.02 |
| 15 | 0 | 12.46 | ± | 0.61 | 2.63 | ± | 0.21 | 1.83 | ± | 0.09 |
|  | 100 | 11.00 | ± | 0.42 | 4.10 | ± | 0.18 | 1.87 | ± | 0.13 |
|  | 200 | 9.04 | ± | 0.48 | 4.25 | ± | 0.23 | 1.81 | ± | 0.07 |
|  | 400 | 8.91 | ± | 0.50 | 4.37 | ± | 0.16 | 2.08 | ± | 0.18 |
| Mar | 0 | 10.91 | ± | 0.53 | 3.63 | ± | 0.04 | 2.26 | ± | 0.03 |
|  | 100 | 11.07 | ± | 0.63 | 4.12 | ± | 0.02 | 2.19 | ± | 0.09 |
|  | 200 | 9.67 | ± | 0.27 | 4.03 | ± | 0.04 | 2.10 | ± | 0.07 |
|  | 400 | 7.84 | ± | 0.10 | 4.13 | ± | 0.20 | 2.32 | ± | 0.09 |

**Table S4** Effects of salinity and variety on leaf fresh weight dry weight ratio (FWDW), succulence (SUC; g leaf FW cm^-2^), cumulative transpiration (Ctrans; Kg H_2_O plant^-1^), water use efficiency (WUE; g DW Kg^-1^ H_2_O), osmotic potential (OP; MPa), and osmotic adjustment (OA; MPa). Var denotes variety; Salt, salinity treatment (0, 100, 200, 400 mM NaCl); Pin, Pin; F16, F-16; Tit, Titicaca; Col, Collana; Pun, Puno; Vik, Vikinga; Pas, Pasankalla; 15, S-15-15; Mar, Marisma. Mean values of five replicates ± standard error

| Var | Salt | *FWDW* | | | *SUC* | | | *Ctrans* | | | *WUE* | | | *OP* | | | *OA* | | |
| --- | --- | --- | --- | --- | --- | --- | --- | --- | --- | --- | --- | --- | --- | --- | --- | --- | --- | --- | --- |
| Pin | 0 | 8.02 | ± | 0.07 | 38.77 | ± | 0.64 | 2.21 | ± | 0.02 | 5.05 | ± | 0.05 | -0.81 | ± | 0.03 |  | - |  |
|  | 100 | 8.25 | ± | 0.10 | 39.26 | ± | 0.35 | 1.77 | ± | 0.03 | 5.88 | ± | 0.15 | -1.37 | ± | 0.06 | 0.53 | ± | 0.02 |
|  | 200 | 7.65 | ± | 0.04 | 40.14 | ± | 0.75 | 1.51 | ± | 0.01 | 5.74 | ± | 0.09 | -1.66 | ± | 0.05 | 0.79 | ± | 0.03 |
|  | 400 | 6.83 | ± | 0.16 | 43.36 | ± | 0.61 | 1.35 | ± | 0.01 | 5.41 | ± | 0.40 | -2.15 | ± | 0.05 | 1.21 | ± | 0.13 |
| F16 | 0 | 9.62 | ± | 0.19 | 50.79 | ± | 1.34 | 1.97 | ± | 0.13 | 4.56 | ± | 0.16 | -0.79 | ± | 0.03 |  | - |  |
|  | 100 | 9.91 | ± | 0.28 | 69.38 | ± | 8.29 | 1.74 | ± | 0.04 | 4.28 | ± | 0.39 | -1.22 | ± | 0.06 | 0.38 | ± | 0.03 |
|  | 200 | 8.74 | ± | 0.10 | 64.66 | ± | 4.00 | 1.56 | ± | 0.03 | 4.71 | ± | 0.20 | -1.46 | ± | 0.05 | 0.59 | ± | 0.03 |
|  | 400 | 7.59 | ± | 0.02 | 67.95 | ± | 5.84 | 1.48 | ± | 0.01 | 4.38 | ± | 0.34 | -1.74 | ± | 0.07 | 0.99 | ± | 0.03 |
| Tit | 0 | 7.70 | ± | 0.14 | 37.19 | ± | 0.57 | 3.02 | ± | 0.24 | 4.18 | ± | 0.28 | -0.92 | ± | 0.03 |  | - |  |
|  | 100 | 7.96 | ± | 0.06 | 37.86 | ± | 0.79 | 2.12 | ± | 0.19 | 5.13 | ± | 0.35 | -1.62 | ± | 0.05 | 0.69 | ± | 0.10 |
|  | 200 | 7.58 | ± | 0.10 | 39.33 | ± | 0.56 | 1.57 | ± | 0.02 | 5.75 | ± | 0.14 | -1.77 | ± | 0.07 | 0.83 | ± | 0.05 |
|  | 400 | 6.63 | ± | 0.05 | 45.54 | ± | 3.94 | 1.30 | ± | 0.01 | 6.00 | ± | 0.14 | -2.31 | ± | 0.04 | 1.29 | ± | 0.07 |
| Col | 0 | 10.69 | ± | 0.29 | 52.82 | ± | 3.68 | 2.00 | ± | 0.07 | 3.42 | ± | 0.37 | -0.85 | ± | 0.04 |  | - |  |
|  | 100 | 10.16 | ± | 0.13 | 55.89 | ± | 2.25 | 1.51 | ± | 0.04 | 4.23 | ± | 0.24 | -1.29 | ± | 0.03 | 0.38 | ± | 0.03 |
|  | 200 | 9.63 | ± | 0.21 | 58.83 | ± | 1.52 | 1.40 | ± | 0.05 | 4.07 | ± | 0.16 | -1.56 | ± | 0.04 | 0.62 | ± | 0.05 |
|  | 400 | 8.03 | ± | 0.09 | 63.30 | ± | 2.39 | 1.31 | ± | 0.01 | 3.73 | ± | 0.23 | -1.94 | ± | 0.05 | 0.96 | ± | 0.04 |
| Pun | 0 | 8.27 | ± | 0.44 | 38.79 | ± | 3.60 | 2.11 | ± | 0.11 | 3.92 | ± | 0.23 | -0.95 | ± | 0.05 |  | - |  |
|  | 100 | 7.86 | ± | 0.11 | 35.30 | ± | 0.42 | 1.70 | ± | 0.02 | 5.24 | ± | 0.22 | -1.56 | ± | 0.04 | 0.56 | ± | 0.03 |
|  | 200 | 7.46 | ± | 0.18 | 36.07 | ± | 0.50 | 1.49 | ± | 0.02 | 5.47 | ± | 0.41 | -1.77 | ± | 0.04 | 0.72 | ± | 0.04 |
|  | 400 | 6.42 | ± | 0.07 | 39.17 | ± | 0.53 | 1.33 | ± | 0.03 | 4.98 | ± | 0.47 | -2.24 | ± | 0.05 | 1.13 | ± | 0.06 |
| Vik | 0 | 9.48 | ± | 0.07 | 36.30 | ± | 0.24 | 2.95 | ± | 0.07 | 3.62 | ± | 0.04 | -0.66 | ± | 0.04 |  | - |  |
|  | 100 | 9.16 | ± | 0.17 | 35.67 | ± | 0.41 | 2.04 | ± | 0.03 | 4.74 | ± | 0.15 | -1.15 | ± | 0.06 | 0.40 | ± | 0.03 |
|  | 200 | 7.82 | ± | 0.17 | 36.36 | ± | 0.57 | 1.64 | ± | 0.02 | 5.11 | ± | 0.20 | -1.48 | ± | 0.07 | 0.65 | ± | 0.04 |
|  | 400 | 7.13 | ± | 0.06 | 38.15 | ± | 0.66 | 1.40 | ± | 0.03 | 4.85 | ± | 0.16 | -1.84 | ± | 0.08 | 1.05 | ± | 0.09 |
| Pas | 0 | 8.57 | ± | 0.14 | 38.44 | ± | 0.62 | 2.77 | ± | 0.07 | 4.44 | ± | 0.15 | -0.84 | ± | 0.03 |  | - |  |
|  | 100 | 8.87 | ± | 0.08 | 40.32 | ± | 0.22 | 1.85 | ± | 0.03 | 5.47 | ± | 0.09 | -1.43 | ± | 0.06 | 0.53 | ± | 0.07 |
|  | 200 | 7.78 | ± | 0.14 | 42.30 | ± | 0.43 | 1.61 | ± | 0.04 | 5.73 | ± | 0.14 | -1.75 | ± | 0.06 | 0.93 | ± | 0.04 |
|  | 400 | 6.68 | ± | 0.06 | 48.31 | ± | 3.40 | 1.47 | ± | 0.02 | 5.57 | ± | 0.23 | -2.42 | ± | 0.05 | 1.67 | ± | 0.08 |
| 15 | 0 | 8.54 | ± | 0.22 | 37.79 | ± | 1.35 | 2.92 | ± | 0.09 | 4.27 | ± | 0.13 | -0.77 | ± | 0.04 |  | - |  |
|  | 100 | 9.02 | ± | 0.26 | 40.05 | ± | 1.34 | 2.20 | ± | 0.07 | 4.98 | ± | 0.07 | -1.30 | ± | 0.07 | 0.49 | ± | 0.05 |
|  | 200 | 7.54 | ± | 0.21 | 38.11 | ± | 0.73 | 1.65 | ± | 0.04 | 5.47 | ± | 0.18 | -1.54 | ± | 0.08 | 0.74 | ± | 0.08 |
|  | 400 | 6.38 | ± | 0.17 | 43.11 | ± | 1.78 | 1.42 | ± | 0.04 | 6.24 | ± | 0.24 | -1.95 | ± | 0.11 | 1.31 | ± | 0.10 |
| Mar | 0 | 9.21 | ± | 0.10 | 33.95 | ± | 0.91 | 2.96 | ± | 0.08 | 3.68 | ± | 0.16 | -0.70 | ± | 0.06 |  | - |  |
|  | 100 | 9.30 | ± | 0.02 | 36.20 | ± | 1.68 | 2.38 | ± | 0.04 | 4.65 | ± | 0.20 | -1.49 | ± | 0.07 | 0.47 | ± | 0.02 |
|  | 200 | 8.40 | ± | 0.16 | 37.02 | ± | 1.05 | 2.01 | ± | 0.02 | 4.81 | ± | 0.16 | -1.72 | ± | 0.05 | 0.87 | ± | 0.02 |
|  | 400 | 7.61 | ± | 0.17 | 43.04 | ± | 1.13 | 1.60 | ± | 0.02 | 4.90 | ± | 0.13 | -2.20 | ± | 0.10 | 1.34 | ± | 0.04 |

**Table S5** Effects of salinity and variety on sodium uptake rate (NaUR; mg Na^+^ g^-1^ root DW day^-1^), chloride uptake rate (ClUR; mg Cl^-^ g^-1^ root DW day^-1^), potassium uptake rate (KUR; mg K^+^ g^-1^ root DW day^-1^), and calcium uptake rate (CaUR; mg Ca^+2^ g^-1^ root DW day^-1^). Var denotes variety; Salt, salinity treatment (0, 100, 200, 400 mM NaCl); Pin, Pin; F16, F-16; Tit, Titicaca; Col, Collana; Pun, Puno; Vik, Vikinga; Pas, Pasankalla; 15, S-15-15; Mar, Marisma. Mean values of five replicates ± standard error

| Var | Salt | *NaUR* | | | *ClUR* | | | *KUR* | | | *CaUR* | | |
| --- | --- | --- | --- | --- | --- | --- | --- | --- | --- | --- | --- | --- | --- |
| Pin | 0 | 1.54 | ± | 0.10 | 0.91 | ± | 0.05 | 40.24 | ± | 0.61 | 10.75 | ± | 0.33 |
|  | 100 | 13.50 | ± | 0.77 | 25.79 | ± | 1.08 | 55.29 | ± | 4.62 | 8.42 | ± | 0.29 |
|  | 200 | 15.63 | ± | 0.88 | 30.18 | ± | 1.72 | 53.07 | ± | 1.95 | 6.29 | ± | 0.26 |
|  | 400 | 18.90 | ± | 0.93 | 35.50 | ± | 1.70 | 56.19 | ± | 1.99 | 5.00 | ± | 0.14 |
| F16 | 0 | 1.83 | ± | 0.04 | 1.51 | ± | 0.07 | 55.45 | ± | 0.95 | 9.67 | ± | 0.31 |
|  | 100 | 11.05 | ± | 0.55 | 25.54 | ± | 0.40 | 70.47 | ± | 1.01 | 7.47 | ± | 0.55 |
|  | 200 | 13.38 | ± | 1.20 | 35.57 | ± | 0.66 | 80.09 | ± | 1.39 | 6.73 | ± | 0.47 |
|  | 400 | 15.19 | ± | 0.45 | 39.00 | ± | 2.45 | 70.37 | ± | 2.34 | 5.62 | ± | 0.15 |
| Tit | 0 | 1.76 | ± | 0.12 | 0.79 | ± | 0.02 | 34.80 | ± | 0.84 | 9.74 | ± | 0.35 |
|  | 100 | 9.17 | ± | 0.39 | 23.08 | ± | 0.13 | 40.65 | ± | 0.54 | 6.81 | ± | 0.31 |
|  | 200 | 13.06 | ± | 0.66 | 26.95 | ± | 0.87 | 41.03 | ± | 1.48 | 5.52 | ± | 0.45 |
|  | 400 | 16.07 | ± | 0.51 | 30.48 | ± | 1.34 | 46.11 | ± | 0.97 | 4.47 | ± | 0.21 |
| Col | 0 | 0.47 | ± | 0.09 | 0.93 | ± | 0.03 | 49.72 | ± | 0.68 | 7.59 | ± | 0.37 |
|  | 100 | 7.27 | ± | 0.77 | 17.31 | ± | 0.59 | 53.62 | ± | 0.74 | 6.24 | ± | 0.29 |
|  | 200 | 9.92 | ± | 1.01 | 21.83 | ± | 1.40 | 46.32 | ± | 3.27 | 4.48 | ± | 0.23 |
|  | 400 | 13.15 | ± | 0.41 | 27.19 | ± | 1.24 | 47.00 | ± | 4.30 | 4.35 | ± | 1.38 |
| Pun | 0 | 1.15 | ± | 0.12 | 0.86 | ± | 0.13 | 47.23 | ± | 5.45 | 10.18 | ± | 0.96 |
|  | 100 | 11.74 | ± | 0.59 | 25.49 | ± | 0.38 | 44.42 | ± | 0.50 | 7.22 | ± | 0.13 |
|  | 200 | 13.38 | ± | 0.51 | 28.80 | ± | 1.46 | 49.39 | ± | 1.17 | 6.30 | ± | 0.54 |
|  | 400 | 16.16 | ± | 1.20 | 32.75 | ± | 1.77 | 52.02 | ± | 1.86 | 4.79 | ± | 0.22 |
| Vik | 0 | 1.49 | ± | 0.02 | 0.70 | ± | 0.07 | 38.91 | ± | 1.12 | 7.99 | ± | 0.40 |
|  | 100 | 7.36 | ± | 0.24 | 17.89 | ± | 1.01 | 41.56 | ± | 1.82 | 5.55 | ± | 0.36 |
|  | 200 | 8.65 | ± | 0.23 | 20.60 | ± | 0.51 | 39.22 | ± | 1.15 | 4.09 | ± | 0.16 |
|  | 400 | 9.53 | ± | 0.60 | 24.17 | ± | 1.70 | 41.92 | ± | 2.34 | 2.75 | ± | 0.15 |
| Pas | 0 | 1.21 | ± | 0.10 | 0.47 | ± | 0.01 | 24.36 | ± | 0.15 | 6.00 | ± | 0.28 |
|  | 100 | 7.68 | ± | 0.04 | 16.20 | ± | 0.19 | 29.84 | ± | 1.80 | 4.21 | ± | 0.07 |
|  | 200 | 9.98 | ± | 0.61 | 18.76 | ± | 0.92 | 27.91 | ± | 1.55 | 3.12 | ± | 0.16 |
|  | 400 | 15.57 | ± | 0.47 | 26.97 | ± | 0.99 | 29.75 | ± | 0.91 | 2.60 | ± | 0.25 |
| 15 | 0 | 1.80 | ± | 0.02 | 0.71 | ± | 0.05 | 29.60 | ± | 1.46 | 7.88 | ± | 0.38 |
|  | 100 | 8.59 | ± | 0.16 | 16.75 | ± | 0.22 | 37.27 | ± | 0.59 | 5.85 | ± | 0.30 |
|  | 200 | 10.56 | ± | 1.38 | 20.56 | ± | 1.73 | 37.98 | ± | 3.46 | 4.46 | ± | 0.16 |
|  | 400 | 14.58 | ± | 1.66 | 28.73 | ± | 3.13 | 42.35 | ± | 2.02 | 4.69 | ± | 0.42 |
| Mar | 0 | 1.41 | ± | 0.11 | 0.86 | ± | 0.01 | 33.96 | ± | 0.11 | 8.32 | ± | 0.19 |
|  | 100 | 10.48 | ± | 0.45 | 23.00 | ± | 1.12 | 37.63 | ± | 0.39 | 6.90 | ± | 0.25 |
|  | 200 | 11.32 | ± | 0.47 | 25.89 | ± | 0.70 | 36.54 | ± | 1.32 | 5.17 | ± | 0.28 |
|  | 400 | 15.95 | ± | 1.43 | 35.36 | ± | 1.35 | 31.44 | ± | 1.39 | 4.17 | ± | 0.07 |

**Table S6** Effects of salinity and variety on sodium shoot to root ratio (SRNa), chloride shoot to root ratio (SRCl), potassium shoot to root ratio (SRK), and calcium shoot to root ratio (SRCa). Var denotes variety; Salt, salinity treatment (0, 100, 200, 400 mM NaCl); Pin, Pin; F16, F-16; Tit, Titicaca; Col, Collana; Pun, Puno; Vik, Vikinga; Pas, Pasankalla; 15, S-15-15; Mar, Marisma. Mean values of five replicates ± standard error

| Var | Salt | *SRNa* | | | *SRCl* | | | *SRK* | | | *SRCa* | | |
| --- | --- | --- | --- | --- | --- | --- | --- | --- | --- | --- | --- | --- | --- |
| Pin | 0 | 0.21 | ± | 0.05 | 0.49 | ± | 0.03 | 1.04 | ± | 0.02 | 0.78 | ± | 0.05 |
|  | 100 | 0.87 | ± | 0.05 | 0.87 | ± | 0.05 | 1.59 | ± | 0.16 | 1.08 | ± | 0.15 |
|  | 200 | 1.04 | ± | 0.15 | 1.33 | ± | 0.15 | 1.83 | ± | 0.17 | 0.99 | ± | 0.11 |
|  | 400 | 1.20 | ± | 0.13 | 2.62 | ± | 0.72 | 2.31 | ± | 0.25 | 0.88 | ± | 0.06 |
| F16 | 0 | 0.14 | ± | 0.01 | 0.81 | ± | 0.06 | 1.10 | ± | 0.08 | 0.49 | ± | 0.03 |
|  | 100 | 0.49 | ± | 0.05 | 1.14 | ± | 0.10 | 2.14 | ± | 0.14 | 0.51 | ± | 0.04 |
|  | 200 | 0.77 | ± | 0.06 | 1.88 | ± | 0.19 | 3.02 | ± | 0.18 | 0.63 | ± | 0.08 |
|  | 400 | 0.63 | ± | 0.11 | 1.81 | ± | 0.27 | 3.01 | ± | 0.40 | 0.44 | ± | 0.02 |
| Tit | 0 | 0.16 | ± | 0.01 | 0.42 | ± | 0.01 | 0.86 | ± | 0.03 | 0.67 | ± | 0.02 |
|  | 100 | 0.49 | ± | 0.03 | 1.07 | ± | 0.10 | 1.36 | ± | 0.06 | 0.80 | ± | 0.03 |
|  | 200 | 0.75 | ± | 0.09 | 1.05 | ± | 0.05 | 1.50 | ± | 0.10 | 0.89 | ± | 0.11 |
|  | 400 | 1.01 | ± | 0.04 | 1.99 | ± | 0.37 | 2.19 | ± | 0.17 | 0.98 | ± | 0.11 |
| Col | 0 | 0.32 | ± | 0.04 | 1.59 | ± | 0.18 | 1.88 | ± | 0.14 | 1.17 | ± | 0.10 |
|  | 100 | 0.33 | ± | 0.08 | 1.18 | ± | 0.20 | 2.33 | ± | 0.05 | 0.73 | ± | 0.02 |
|  | 200 | 0.53 | ± | 0.20 | 1.46 | ± | 0.19 | 2.35 | ± | 0.20 | 0.66 | ± | 0.10 |
|  | 400 | 1.00 | ± | 0.08 | 2.68 | ± | 0.36 | 3.10 | ± | 0.05 | 1.03 | ± | 0.25 |
| Pun | 0 | 0.31 | ± | 0.07 | 0.86 | ± | 0.13 | 1.31 | ± | 0.10 | 1.08 | ± | 0.31 |
|  | 100 | 0.55 | ± | 0.08 | 0.83 | ± | 0.01 | 1.31 | ± | 0.07 | 0.91 | ± | 0.15 |
|  | 200 | 0.61 | ± | 0.02 | 0.94 | ± | 0.02 | 1.62 | ± | 0.06 | 0.72 | ± | 0.03 |
|  | 400 | 0.87 | ± | 0.08 | 1.85 | ± | 0.38 | 2.36 | ± | 0.22 | 0.94 | ± | 0.04 |
| Vik | 0 | 0.16 | ± | 0.01 | 0.64 | ± | 0.09 | 1.63 | ± | 0.04 | 0.86 | ± | 0.06 |
|  | 100 | 0.39 | ± | 0.08 | 1.23 | ± | 0.29 | 2.24 | ± | 0.19 | 0.77 | ± | 0.10 |
|  | 200 | 0.46 | ± | 0.03 | 1.44 | ± | 0.26 | 2.43 | ± | 0.05 | 0.61 | ± | 0.06 |
|  | 400 | 0.53 | ± | 0.02 | 2.70 | ± | 0.42 | 3.48 | ± | 0.17 | 0.72 | ± | 0.03 |
| Pas | 0 | 0.02 | ± | 0.00 | 0.28 | ± | 0.03 | 1.08 | ± | 0.06 | 0.44 | ± | 0.03 |
|  | 100 | 0.20 | ± | 0.01 | 0.99 | ± | 0.26 | 2.20 | ± | 0.35 | 0.71 | ± | 0.10 |
|  | 200 | 0.25 | ± | 0.01 | 0.98 | ± | 0.17 | 2.18 | ± | 0.02 | 0.49 | ± | 0.03 |
|  | 400 | 0.51 | ± | 0.04 | 1.88 | ± | 0.41 | 2.59 | ± | 0.03 | 0.49 | ± | 0.03 |
| 15 | 0 | 0.18 | ± | 0.01 | 0.45 | ± | 0.04 | 0.89 | ± | 0.03 | 0.53 | ± | 0.02 |
|  | 100 | 0.47 | ± | 0.07 | 0.94 | ± | 0.07 | 1.48 | ± | 0.23 | 0.95 | ± | 0.09 |
|  | 200 | 0.51 | ± | 0.06 | 1.81 | ± | 0.40 | 1.91 | ± | 0.15 | 0.83 | ± | 0.20 |
|  | 400 | 0.63 | ± | 0.02 | 2.14 | ± | 0.59 | 1.63 | ± | 0.18 | 0.65 | ± | 0.09 |
| Mar | 0 | 0.32 | ± | 0.05 | 0.59 | ± | 0.08 | 1.07 | ± | 0.04 | 1.07 | ± | 0.14 |
|  | 100 | 0.87 | ± | 0.05 | 1.40 | ± | 0.19 | 1.18 | ± | 0.04 | 1.15 | ± | 0.10 |
|  | 200 | 0.71 | ± | 0.03 | 1.15 | ± | 0.03 | 1.40 | ± | 0.05 | 0.68 | ± | 0.03 |
|  | 400 | 1.01 | ± | 0.02 | 1.67 | ± | 0.06 | 1.50 | ± | 0.12 | 0.79 | ± | 0.06 |

**Table S7** Effects of salinity and variety on sodium leaf to stem ratio (LSNa), chloride leaf to stem ratio (LSCl), potassium leaf to stem ratio (LSK), and calcium leaf to stem ratio (LSCa). Var denotes variety; Salt, salinity treatment (0, 100, 200, 400 mM NaCl); Pin, Pin; F16, F-16; Tit, Titicaca; Col, Collana; Pun, Puno; Vik, Vikinga; Pas, Pasankalla; 15, S-15-15; Mar, Marisma. Mean values of five replicates ± standard error

| Var | Salt | *LSNa* | | | *LSCl* | | | *LSK* | | | *LSCa* | | |
| --- | --- | --- | --- | --- | --- | --- | --- | --- | --- | --- | --- | --- | --- |
| Pin | 0 | 1.32 | ± | 0.21 | 0.27 | ± | 0.01 | 0.64 | ± | 0.04 | 1.86 | ± | 0.03 |
|  | 100 | 1.00 | ± | 0.18 | 0.38 | ± | 0.03 | 0.43 | ± | 0.08 | 1.96 | ± | 0.36 |
|  | 200 | 1.27 | ± | 0.05 | 0.42 | ± | 0.02 | 0.50 | ± | 0.01 | 2.50 | ± | 0.04 |
|  | 400 | 1.05 | ± | 0.03 | 0.55 | ± | 0.03 | 0.58 | ± | 0.01 | 2.67 | ± | 0.04 |
| F16 | 0 | 1.15 | ± | 0.12 | 0.56 | ± | 0.08 | 0.56 | ± | 0.01 | 2.57 | ± | 0.20 |
|  | 100 | 0.52 | ± | 0.05 | 0.39 | ± | 0.02 | 0.59 | ± | 0.01 | 3.33 | ± | 0.29 |
|  | 200 | 0.56 | ± | 0.01 | 0.52 | ± | 0.04 | 0.65 | ± | 0.02 | 3.37 | ± | 0.11 |
|  | 400 | 0.53 | ± | 0.02 | 0.72 | ± | 0.06 | 0.75 | ± | 0.03 | 3.52 | ± | 0.06 |
| Tit | 0 | 1.63 | ± | 0.11 | 0.44 | ± | 0.06 | 0.58 | ± | 0.00 | 1.61 | ± | 0.08 |
|  | 100 | 0.98 | ± | 0.05 | 0.38 | ± | 0.01 | 0.56 | ± | 0.00 | 2.57 | ± | 0.16 |
|  | 200 | 0.95 | ± | 0.04 | 0.44 | ± | 0.01 | 0.51 | ± | 0.02 | 2.58 | ± | 0.04 |
|  | 400 | 0.75 | ± | 0.09 | 0.50 | ± | 0.01 | 0.61 | ± | 0.03 | 2.27 | ± | 0.05 |
| Col | 0 | 1.17 | ± | 0.22 | 0.39 | ± | 0.04 | 0.69 | ± | 0.01 | 1.56 | ± | 0.17 |
|  | 100 | 2.04 | ± | 0.54 | 0.41 | ± | 0.03 | 0.59 | ± | 0.01 | 2.32 | ± | 0.09 |
|  | 200 | 1.44 | ± | 0.41 | 0.54 | ± | 0.02 | 0.60 | ± | 0.04 | 2.98 | ± | 0.25 |
|  | 400 | 1.85 | ± | 0.02 | 0.63 | ± | 0.03 | 0.56 | ± | 0.10 | 2.02 | ± | 0.88 |
| Pun | 0 | 2.44 | ± | 0.09 | 0.47 | ± | 0.09 | 0.60 | ± | 0.04 | 1.48 | ± | 0.17 |
|  | 100 | 1.51 | ± | 0.20 | 0.55 | ± | 0.05 | 0.52 | ± | 0.04 | 2.17 | ± | 0.12 |
|  | 200 | 1.23 | ± | 0.03 | 0.56 | ± | 0.05 | 0.45 | ± | 0.02 | 2.49 | ± | 0.13 |
|  | 400 | 1.11 | ± | 0.05 | 0.54 | ± | 0.03 | 0.54 | ± | 0.04 | 2.61 | ± | 0.18 |
| Vik | 0 | 1.80 | ± | 0.08 | 0.46 | ± | 0.02 | 0.63 | ± | 0.04 | 2.01 | ± | 0.04 |
|  | 100 | 1.19 | ± | 0.09 | 0.68 | ± | 0.08 | 0.49 | ± | 0.01 | 3.14 | ± | 0.07 |
|  | 200 | 1.08 | ± | 0.03 | 0.53 | ± | 0.02 | 0.56 | ± | 0.00 | 3.23 | ± | 0.08 |
|  | 400 | 0.88 | ± | 0.04 | 0.64 | ± | 0.08 | 0.55 | ± | 0.02 | 3.30 | ± | 0.22 |
| Pas | 0 | 3.81 | ± | 0.66 | 0.59 | ± | 0.03 | 0.60 | ± | 0.03 | 1.87 | ± | 0.01 |
|  | 100 | 1.57 | ± | 0.11 | 0.66 | ± | 0.01 | 0.49 | ± | 0.02 | 3.52 | ± | 0.24 |
|  | 200 | 1.49 | ± | 0.22 | 0.79 | ± | 0.02 | 0.48 | ± | 0.02 | 3.68 | ± | 0.09 |
|  | 400 | 1.81 | ± | 0.07 | 0.98 | ± | 0.03 | 0.50 | ± | 0.01 | 3.58 | ± | 0.09 |
| 15 | 0 | 1.91 | ± | 0.25 | 0.52 | ± | 0.06 | 0.49 | ± | 0.04 | 1.38 | ± | 0.11 |
|  | 100 | 1.57 | ± | 0.08 | 0.53 | ± | 0.09 | 0.48 | ± | 0.00 | 2.24 | ± | 0.05 |
|  | 200 | 1.02 | ± | 0.06 | 0.49 | ± | 0.11 | 0.48 | ± | 0.02 | 2.46 | ± | 0.36 |
|  | 400 | 0.94 | ± | 0.07 | 0.59 | ± | 0.03 | 0.51 | ± | 0.00 | 2.18 | ± | 0.17 |
| Mar | 0 | 1.77 | ± | 0.06 | 0.37 | ± | 0.03 | 0.52 | ± | 0.01 | 1.43 | ± | 0.07 |
|  | 100 | 1.02 | ± | 0.10 | 0.48 | ± | 0.04 | 0.45 | ± | 0.05 | 2.23 | ± | 0.20 |
|  | 200 | 0.90 | ± | 0.06 | 0.54 | ± | 0.02 | 0.48 | ± | 0.02 | 2.55 | ± | 0.17 |
|  | 400 | 0.87 | ± | 0.08 | 0.62 | ± | 0.06 | 0.47 | ± | 0.03 | 2.57 | ± | 0.31 |

**Table S8** Effects of salinity and variety on superoxide dismutase (SOD; U mg^-1^ protein), catalase (CAT; μmol H_2_O_2_ min^-1^ mg^-1^ protein), glutathione reductase (GR; nmol NADPH min^-1^ mg^-1^ protein), dehydroascorbate reductase (DHAR; nmol ASA min^-1^ mg^-1^ protein), reduced ascorbate (ASA; μmol g^-1^ DW), monodehydroascorbate reductase (MDHAR; nmol NADH min^-1^ mg protein^-1^) and reduced glutathione (GSH; μmol g^-1^ DW). Var denotes variety; Salt, salinity treatment (0, 100, 200, 400 mM NaCl); Pin, Pin; F16, F-16; Tit, Titicaca; Col, Collana; Pun, Puno; Vik, Vikinga; Pas, Pasankalla; 15, S-15-15; Mar, Marisma. Mean values of five replicates ± standard error

| Var | Salt | *SOD* | | | *CAT* | | | *GR* | | | *DHAR* | | | *ASA* | | | *MDHAR* | | | *GSH* | | |
| --- | --- | --- | --- | --- | --- | --- | --- | --- | --- | --- | --- | --- | --- | --- | --- | --- | --- | --- | --- | --- | --- | --- |
| Pin | 0 | 19.87 | ± | 2.14 | 90.82 | ± | 4.79 | 74.46 | ± | 6.13 | 77.98 | ± | 5.84 | 10.80 | ± | 1.05 | 57.04 | ± | 9.57 | 1187 | ± | 157.40 |
|  | 100 | 24.21 | ± | 2.81 | 137.38 | ± | 11.37 | 82.90 | ± | 7.23 | 71.11 | ± | 4.41 | 8.40 | ± | 0.42 | 52.39 | ± | 4.25 | 1387 | ± | 125.71 |
|  | 200 | 22.05 | ± | 3.48 | 130.43 | ± | 6.47 | 77.62 | ± | 3.27 | 74.46 | ± | 9.92 | 7.45 | ± | 0.46 | 58.50 | ± | 6.53 | 1378 | ± | 42.08 |
|  | 400 | 19.12 | ± | 2.60 | 137.13 | ± | 4.65 | 78.83 | ± | 3.43 | 80.00 | ± | 8.28 | 7.30 | ± | 0.24 | 58.56 | ± | 2.76 | 1380 | ± | 95.86 |
| F16 | 0 | 21.46 | ± | 2.31 | 137.43 | ± | 11.32 | 89.87 | ± | 1.59 | 120.21 | ± | 5.50 | 12.94 | ± | 1.76 | 80.70 | ± | 9.36 | 1312 | ± | 155.13 |
|  | 100 | 20.00 | ± | 2.75 | 136.98 | ± | 14.34 | 83.72 | ± | 2.48 | 114.73 | ± | 6.52 | 9.58 | ± | 0.53 | 69.14 | ± | 2.81 | 1126 | ± | 98.84 |
|  | 200 | 24.66 | ± | 2.81 | 163.26 | ± | 12.87 | 92.56 | ± | 3.66 | 113.28 | ± | 4.92 | 9.24 | ± | 0.56 | 69.44 | ± | 2.61 | 1433 | ± | 97.82 |
|  | 400 | 27.66 | ± | 5.06 | 162.88 | ± | 27.11 | 95.72 | ± | 4.69 | 127.87 | ± | 2.41 | 9.50 | ± | 0.56 | 70.00 | ± | 1.27 | 1405 | ± | 85.61 |
| Tit | 0 | 23.95 | ± | 5.15 | 118.23 | ± | 14.52 | 74.26 | ± | 4.55 | 84.59 | ± | 12.4 | 20.58 | ± | 2.77 | 73.15 | ± | 12.73 | 1522 | ± | 131.05 |
|  | 100 | 29.88 | ± | 4.62 | 158.08 | ± | 25.63 | 85.95 | ± | 3.78 | 83.10 | ± | 11.3 | 11.68 | ± | 1.61 | 61.80 | ± | 4.86 | 1729 | ± | 45.25 |
|  | 200 | 31.12 | ± | 3.87 | 168.78 | ± | 18.91 | 79.07 | ± | 3.65 | 79.97 | ± | 8.66 | 11.42 | ± | 1.26 | 76.67 | ± | 9.94 | 1779 | ± | 168.77 |
|  | 400 | 33.47 | ± | 5.56 | 154.31 | ± | 15.12 | 82.54 | ± | 7.68 | 80.41 | ± | 5.94 | 8.99 | ± | 0.72 | 70.55 | ± | 5.21 | 1574 | ± | 127.59 |
| Col | 0 | 24.69 | ± | 3.62 | 143.42 | ± | 21.71 | 82.34 | ± | 3.84 | 112.03 | ± | 5.88 | 12.01 | ± | 1.28 | 82.94 | ± | 6.62 | 1393 | ± | 128.09 |
|  | 100 | 27.05 | ± | 4.24 | 158.33 | ± | 20.15 | 82.07 | ± | 2.88 | 104.92 | ± | 8.89 | 11.00 | ± | 1.23 | 77.90 | ± | 8.36 | 1797 | ± | 188.26 |
|  | 200 | 31.29 | ± | 2.26 | 152.82 | ± | 20.92 | 87.28 | ± | 3.90 | 124.94 | ± | 9.72 | 12.01 | ± | 1.16 | 81.43 | ± | 4.30 | 1615 | ± | 86.39 |
|  | 400 | 31.14 | ± | 2.57 | 148.72 | ± | 13.68 | 87.06 | ± | 2.83 | 123.68 | ± | 8.21 | 10.53 | ± | 0.98 | 82.22 | ± | 5.28 | 1663 | ± | 97.32 |
| Pun | 0 | 26.22 | ± | 2.68 | 112.43 | ± | 15.14 | 69.60 | ± | 3.24 | 89.59 | ± | 6.08 | 13.00 | ± | 1.62 | 79.46 | ± | 11.49 | 1658 | ± | 135.21 |
|  | 100 | 32.36 | ± | 3.35 | 141.34 | ± | 12.84 | 71.55 | ± | 4.78 | 101.12 | ± | 8.78 | 10.86 | ± | 0.48 | 79.37 | ± | 5.62 | 1646 | ± | 96.44 |
|  | 200 | 24.73 | ± | 1.82 | 177.92 | ± | 23.38 | 85.14 | ± | 4.18 | 94.27 | ± | 6.94 | 10.28 | ± | 0.44 | 86.74 | ± | 8.98 | 1955 | ± | 83.45 |
|  | 400 | 28.69 | ± | 3.55 | 155.88 | ± | 16.26 | 79.14 | ± | 2.62 | 110.89 | ± | 6.03 | 9.77 | ± | 0.40 | 88.87 | ± | 7.71 | 1805 | ± | 45.01 |
| Vik | 0 | 33.19 | ± | 8.40 | 119.65 | ± | 9.73 | 90.65 | ± | 2.95 | 80.39 | ± | 7.29 | 10.93 | ± | 0.73 | 59.56 | ± | 6.91 | 1401 | ± | 38.32 |
|  | 100 | 47.81 | ± | 6.95 | 173.32 | ± | 15.36 | 91.79 | ± | 5.33 | 75.14 | ± | 7.69 | 9.05 | ± | 0.39 | 57.24 | ± | 6.29 | 1647 | ± | 79.35 |
|  | 200 | 44.88 | ± | 11.0 | 166.72 | ± | 17.30 | 98.06 | ± | 5.59 | 85.65 | ± | 8.03 | 8.09 | ± | 0.31 | 60.55 | ± | 4.70 | 1470 | ± | 182.44 |
|  | 400 | 37.82 | ± | 2.78 | 157.83 | ± | 15.50 | 100.12 | ± | 2.92 | 91.46 | ± | 10.0 | 8.09 | ± | 0.53 | 63.02 | ± | 6.57 | 1547 | ± | 106.79 |
| Pas | 0 | 21.56 | ± | 3.19 | 106.35 | ± | 18.11 | 75.18 | ± | 2.83 | 87.21 | ± | 2.55 | 17.27 | ± | 1.31 | 74.52 | ± | 5.18 | 1677 | ± | 161.82 |
|  | 100 | 29.12 | ± | 4.50 | 112.01 | ± | 8.60 | 81.45 | ± | 3.79 | 93.72 | ± | 4.43 | 14.54 | ± | 0.92 | 70.71 | ± | 2.79 | 1523 | ± | 231.42 |
|  | 200 | 25.43 | ± | 3.71 | 127.98 | ± | 5.61 | 87.41 | ± | 4.45 | 104.42 | ± | 9.97 | 12.29 | ± | 0.79 | 79.93 | ± | 5.82 | 1613 | ± | 154.84 |
|  | 400 | 26.96 | ± | 8.06 | 125.34 | ± | 5.28 | 98.93 | ± | 1.79 | 104.25 | ± | 5.48 | 13.53 | ± | 0.60 | 71.56 | ± | 3.88 | 1553 | ± | 116.83 |
| 15 | 0 | 21.78 | ± | 2.09 | 95.77 | ± | 6.16 | 68.58 | ± | 5.86 | 109.21 | ± | 12.9 | 13.50 | ± | 1.14 | 65.95 | ± | 3.90 | 1245 | ± | 69.25 |
|  | 100 | 26.82 | ± | 3.73 | 107.86 | ± | 10.45 | 70.60 | ± | 1.13 | 89.60 | ± | 7.52 | 9.98 | ± | 0.50 | 61.67 | ± | 4.90 | 1366 | ± | 172.29 |
|  | 200 | 25.47 | ± | 3.81 | 139.51 | ± | 9.54 | 75.98 | ± | 5.78 | 100.64 | ± | 6.08 | 10.41 | ± | 0.28 | 68.73 | ± | 3.63 | 1723 | ± | 164.02 |
|  | 400 | 28.73 | ± | 4.48 | 115.28 | ± | 7.99 | 70.66 | ± | 4.38 | 102.08 | ± | 12.2 | 10.52 | ± | 0.72 | 62.69 | ± | 6.21 | 1318 | ± | 77.94 |
| Mar | 0 | 24.67 | ± | 4.18 | 101.80 | ± | 4.90 | 78.00 | ± | 3.33 | 73.63 | ± | 2.15 | 13.15 | ± | 0.72 | 62.26 | ± | 7.53 | 1712 | ± | 182.87 |
|  | 100 | 30.79 | ± | 4.45 | 124.10 | ± | 8.68 | 72.20 | ± | 4.72 | 64.95 | ± | 3.48 | 9.97 | ± | 0.66 | 62.33 | ± | 6.03 | 1428 | ± | 203.85 |
|  | 200 | 25.49 | ± | 2.62 | 136.33 | ± | 5.45 | 77.33 | ± | 4.23 | 73.78 | ± | 3.81 | 9.29 | ± | 0.41 | 67.27 | ± | 3.33 | 1431 | ± | 145.22 |
|  | 400 | 32.05 | ± | 3.14 | 144.22 | ± | 6.05 | 83.48 | ± | 3.60 | 82.77 | ± | 7.18 | 8.55 | ± | 0.49 | 64.76 | ± | 7.25 | 1455 | ± | 173.74 |
